# Distinct Effects of Respiratory Viral Infection Models on miR-149-5p, IL-6 and p63 Expression in BEAS-2B and A549 Epithelial Cells

**DOI:** 10.3390/cells13110919

**Published:** 2024-05-26

**Authors:** Nafeesa Shahdab, Christopher Ward, Philip M. Hansbro, Stephen Cummings, John S. Young, Fatemeh Moheimani

**Affiliations:** 1 National Horizons Centre, School of Health and Life Sciences, Teesside University, Middlesbrough TS1 3BX, UK; n.shahdab@tees.ac.uk (N.S.); s.cummings@tees.ac.uk (S.C.); j.young@tees.ac.uk (J.S.Y.); 2 Translational and Clinical Research Institute, Newcastle University, Newcastle upon Tyne NE1 7RU, UK; chris.ward@newcastle.ac.uk; 3 Centre for Inflammation, Centenary Institute and University of Technology Sydney, Faculty of Science, School of Life Sciences, Sydney 2007, Australia; philip.hansbro@uts.edu.au; 4 Department of Life Sciences, Manchester Metropolitan University, Manchester M15 6BH, UK

**Keywords:** miR-149-5p, epithelial cells, IL-6, p63, poly (I:C), SARS-CoV-2 spike protein

## Abstract

Respiratory viruses cause airway inflammation, resulting in epithelial injury and repair. miRNAs, including miR-149-5p, regulate different pathological conditions. We aimed to determine how miR-149-5p functions in regulating pro-inflammatory IL-6 and p63, key regulators of airway epithelial wound repair, in response to viral proteins in bronchial (BEAS-2B) and alveolar (A549) epithelial cells. BEAS-2B or A549 cells were incubated with poly (I:C, 0.5 µg/mL) for 48 h or SARS-CoV-2 spike protein-1 or 2 subunit (S1 or S2, 1 μg/mL) for 24 h. miR-149-5p was suppressed in BEAS-2B challenged with poly (I:C), correlating with IL-6 and p63 upregulation. miR-149-5p was down-regulated in A549 stimulated with poly (I:C); IL-6 expression increased, but p63 protein levels were undetectable. miR-149-5p remained unchanged in cells exposed to S1 or S2, while S1 transfection increased IL-6 expression in BEAS-2B cells. Ectopic over-expression of miR-149-5p in BEAS-2B cells suppressed *IL-6* and *p63* mRNA levels and inhibited poly (I:C)-induced *IL-6* and *p63* mRNA expressions. miR-149-5p directly suppressed *IL-6* mRNA in BEAS-2B cells. Hence, BEAS-2B cells respond differently to poly (I:C), S1 or S2 compared to A549 cells. Thus, miR-149-5p dysregulation may be involved in poly (I:C)-stimulated but not S1- or S2-stimulated increased IL-6 production and p63 expression in BEAS-2B cells.

## 1. Introduction

Respiratory viruses are the most common cause of respiratory infections in humans, leading to significant morbidity and mortality worldwide [1,2]. In recent years, respiratory viruses, including influenza A virus and severe acute respiratory syndrome coronavirus 2 (SARS-CoV-2), have emerged as serious threats to public health [3,4]. The airway epithelium is the first line of defence against inhaled respiratory viruses [5,6]. Bronchial epithelial cells line the large airways and are more exposed to inhaled pathogens than alveolar epithelial cells; hence, they have evolved to be more responsive to viral infection [7]. Bronchial cells also express a higher density of viral receptors and trigger a stronger inflammatory response [8,9,10]. Immortalised cells, including human bronchial (BEAS-2B) and alveolar (A549) epithelial cell lines, are commonly used to investigate respiratory viral responses in epithelial cells [11]. Airway epithelial cells express pattern-recognition receptors, including the Toll-like receptor (TLR) family [12]. TLRs recognise virus-generated pathogen-associated recognition molecular patterns (PAMPs) and initiate inflammatory host responses [12].

Polyinosinic:polycytidylic acid (poly (I:C)) is a synthetic double-stranded RNA (dsRNA) representative of the viral dsRNA PAMPs generated as the by-product of viral replication [13,14]. Poly (I:C) is recognised by TLR3 and mediates Toll-interleukin (IL-)1 receptor domain-containing adapter protein-dependent signalling to activate interferon regulatory factor-3 (IRF3) and induces the production of type I interferons (IFNs) [13]. TLR3 further recruits tumour necrosis factor receptor (TNFR)-associated factor-6 (TRAF6) and receptor-interacting protein to activate nuclear factor kappa B (NF-κB), which regulates the production of inflammatory cytokines [13]. There are different receptors and pathways responsible for the pro-inflammatory responses of epithelial cells to SARS-CoV-2 [5,15,16,17,18]. The SARS-CoV-2 spike protein S1 subunit possesses PAMP-like properties [17]. During SARS-CoV-2 entry into airway epithelial cells, the S1 subunit is cleaved and recognised by TLR2. TLR2 activates NF-κB and generates an inflammatory response in epithelial cells [15,16,17].

IL-6 is a major pro-inflammatory cytokine produced by bronchial epithelial cells in response to poly (I:C) or the S1 subunit [18,19,20]. IL-6 induces multiple signalling events that drive pathogenic inflammation and immune responses [21]. Moreover, bronchial epithelial cells incubated with poly (I:C) exhibit diminished injury repair capacity [22]. ΔNp63α, an isoform of tumour protein 63 (p63), is an important transcription factor expressed in basal airway epithelial cells. ΔNp63α regulates the expression of key genes involved in wound repair, including β-catenin, epidermal growth factor receptor (EGFR) and Jagged1 [23]. The effect of respiratory viral infection on p63 in epithelial cells has not yet been fully established.

microRNAs (miRNAs) are non-coding RNAs composed of ~22 nucleotides that regulate post-transcriptional gene silencing [24,25,26]. miRNAs are key mediators of crosstalk between respiratory viruses and host cells [27,28,29]. miR-149-5p orchestrates regulatory roles in several cellular and pathological processes, including the cell cycle, proliferation, apoptosis and inflammation [30,31]. miR-149-5p targets IL-6 in gastric stroma fibroblasts and regulates the cross talk between tumour cells and the tumour stroma [32]. Different miRNA-target prediction databases (STarMir, miRDB and TargetScan) predicted that the 3′UTRs of *IL-6* and *p63* mRNA contains a potential miR-149-5p response element [33,34,35]. Hence, we aimed to investigate the function of miR-149-5p in airway epithelial cells after exposure to respiratory viral PAMPs, including poly (I:C) or SARS-CoV-2 spike protein 1 or 2 subunit (S1 or S2).

Based on background literatures and miRNA platform databases, we hypothesised that miR-149-5p expression is downregulated in BEAS-2B cells and A549 cells exposed to poly (I:C), S1 or S2, leading to upregulations of its potential targets, IL-6 and p63. We showed that miR-149-5p is involved in the regulation of IL-6 and p63 in BEAS-2B cells exposed to poly (I:C) but not S1 or S2. Although miR-149-5p was downregulated in A549 cells incubated with poly (I:C), IL-6 expression increased at lower levels compared to BEAS-2B cells, and ΔNp63α was undetectable. Interestingly, in cells exposed to S1 or S2, the level of miR-149-5p remained unchanged, while IL-6 expressions increased. BEAS-2B cells respond differently to poly (I:C), S1 or S2 compared with A549 cells. Thus, the effect of miR-149-5p is cell type and stimulus specific. Poly (I:C)-associated miR-149-5p dysregulation may potentiate increased IL-6 and p63 expression in bronchial epithelial cells.

## 2. Materials and Methods

### 2.1. Cell Culture and Treatments

This study was approved by Teesside University Research Ethics Committee. Human bronchial simian virus (SV40)-transformed (BEAS-2B) [36] and type II alveolar epithelial carcinoma (A549) cells were purchased from the American Type Culture Collection (ATCC, Manassas, VA, USA) [37]. BEAS-2B cells were maintained in a bronchial epithelium basal growth medium (BEGM) (Lonza, Basel, Switzerland) containing a bronchial epithelial basal medium (BEBM), SingleQuot bullet kit (bovine pituitary extract, hydrocortisone, human epidermal growth factor, epinephrine, transferrin, insulin, retinoic acid and triiodothyronine), amphotericin (2.5 μg/mL), penicillin (100 U/mL) and streptomycin (100 μg/mL) (Gibco, Paisley, UK) [36]. A549 cells were maintained in Kaighn’s modification of Ham’s F-12 (F-12K) medium (ATCC, Manassas, VA, USA) with foetal bovine serum (FBS, 10%) (Merck, Rahway, NJ, USA), penicillin (100 U/mL) and streptomycin (100 μg/mL) [37].

#### 2.1.1. Poly (I:C) Incubation

BEAS-2B or A549 cells were cultured at 1 × 10^5^ cells per well in 24-well plates in a BEGM or F-12K medium (FBS, 10%), respectively. Cells at 70% confluency were washed with Dulbecco’s Phosphate-Buffered Saline (D-PBS) and incubated with a BEBM or F-12K medium containing insulin-transferrin-sodium selenite and linoleic-bovine serum albumin (ITS+1, 1%) (Sigma-Aldrich, Gillingham, UK) for 1 h. Thereafter, baseline (0 h) cell culture supernatant and cell lysate samples were harvested. Other cells were incubated in a BEBM or F-12K medium containing ITS+1 (1%) with poly (I:C) for 6, 8, 24 or 48 h.

#### 2.1.2. Transfection of Cells with S1 or S2

BEAS-2B or A549 cells were cultured at 1 × 10^5^ cells per well in 24-well plates in a BEGM or F-12K medium (FBS, 10%). Cells at 70% confluency were washed with D-PBS, and a BEBM or F-12K medium was added to BEAS-2B or A549 cells, respectively, for 1 h. After 1 h of incubation, cell culture supernatants and cell lysates were harvested at the baseline (0 h). For transfection, cells were incubated in a BEBM or F-12K medium containing the Pierce Protein Transfection Reagent (10 μL, Thermo Fisher, Loughborough, UK) without or with S1 or S2 (1 μg, RayBiotech, Peachtree Corners, GA, USA) at 37 °C [38]. After 4 h of transfection, the BEGM or F-12K medium containing FBS (20%) was added to the cells, and cell culture supernatants and lysate samples were collected. Subsequent samples were collected at 8 and 24 h post-transfection. To determine transfection efficiency, cells were transfected with PierceTM Recombinant GFP (1 μg) (Thermo Fisher). After 4 h of transfection, the cells were washed with PBS, and images were obtained using a Leica DMi8 S Inverted Microscope Solution (Leica Microsystems, Linford Wood, UK Ltd.).

### 2.2. Transfection of miRNA Mimic/Inhibitor

BEAS-2B cells were cultured at 1 × 10^5^ cells per well in a 24-well plate in a BEGM medium. Cells at 70% confluency were transfected, as described before [28], with miR-149-5p mimic, miRNA mimic negative control (mirVana, Invitrogen, Loughborough, UK), miR-149-5p inhibitor or miRNA inhibitor negative control (mirVana) at a total concentration of 5 nM using the Lipofectamine^®^ RNAiMAX Transfection Reagent (Invitrogen) in an Opti-MEM medium (Gibco).

### 2.3. Cell Viability

Epithelial cells were incubated with poly (I:C) (0.01–10 μg/mL for 24 and 48 h) or transfected with S1 or S2 (1 μg/mL) for 24 h. Cell viability was assessed by measuring lactate dehydrogenase (LDH) release [28] at each timepoint using a SpectraMax iD5 Multi-Mode Microplate Reader (Molecular Devices, Wokingham, UK).

### 2.4. Real-Time Polymerase Chain Reaction

miRNA and mRNA were extracted using miRNeasy Mini Kits (Qiagen, Manchester, UK). miRNA (100 ng) or mRNA (200 ng) were reverse transcribed to cDNA using Taqman MicroRNA Reverse Transcription reagent or High-Capacity cDNA Reverse Transcription Kits (Applied Biosystems, Warrington, UK), respectively. Predeveloped primer/probe sets, including TaqMan miRNA assays and TaqMan gene expression assays, were purchased from Applied Biosystems. Quantitative real-time (RT-q)PCR was performed according to the manufacturer’s instructions using a CFX96 Touch Real-Time PCR Detection System (Bio-Rad, Watford, UK) and analysed using the CFX MaestroTM Software (version 4.1.2433.1219, Bio-Rad). RNU44 or 18S ribosomal RNA (Applied Biosystems) was used as the reference miRNA or mRNA, respectively [28]. The cycle threshold (Ct) value was normalised to that of RNU44 or 18S rRNA genes (∆Ct). Data are expressed as 2^−∆∆Ct^ relative to the negative control or media control at baseline [28].

### 2.5. Immunoblotting

Cells were lysed in a RIPA buffer. The protein content of supernatants was determined using the BCA protein assay (Thermo Scientific, Loughborough, UK). All proteins were standardised to 10 μg and resolved by SDS-PAGE. Proteins were then transferred onto nitrocellulose membranes for detection of ΔNp63α, TLR3 and TLR2. GAPDH was used as a loading control for proteins in cell lysates [28]. The following antibodies were used for immunoblotting: purified anti-p63 (ΔN) antibody (Cell Signaling Technology, 67825, Danvers, MA, USA), anti-TLR3 antibody (R&D systems, Bio-techne, AF1487, Abingdon, UK), anti-TLR2 antibody (R&D systems, Bio-techne, AF2616), anti-GAPDH antibody (Abcam, ab9483, Cambridge, UK), donkey anti-goat IgG (HRP, Abcam, ab205723) and goat anti-rabbit IgG (HRP, Abcam, ab6721). Densitometry quantification was performed using Image Lab Software (version 6.0.1) [39]. Values are expressed as the protein/GAPDH ratio and normalised to negative controls or media controls at the baseline.

### 2.6. Enzyme-Linked Immunosorbent Assay

IL-6 release in the cell culture supernatants was analysed using Human IL-6 DuoSet ELISA kits (R&D Systems, Minneapolis, MN, USA) according to the manufacturer’s instructions. All measurements were performed in duplicate using a SpectraMax iD5 Multi-Mode Microplate Reader. The concentration of IL-6 was obtained from the four-parameter logistic (4-PL) curve fit using SoftMax Pro Software (version 7.1).

### 2.7. Luciferase Reporter Assay

To verify whether IL-6 or p63 is a direct target of miR-149-5p, a wild-type or mutant 3′-UTR fragment of *IL-6* (position 1 to 425, 425 base pairs) or *p63* (position 2357 to 2774, 417 base pairs) was subcloned into the pmir-GLO dual-luciferase miRNA target expression vector (Promega, Chilworth, UK) downstream of the firefly luciferase open reading frame (GenScript, Oxford, UK). BEAS-2B cells were cultured in 96-well clear-bottom plates (Thermo Scientific) at a density of 4 × 10^4^ cells per well in a BEGM medium and maintained until 70% confluent. BEAS-2B cells were co-transfected with the miR-149-5p mimic or the negative control for the mimic (0.03 μM) and the pmir-GLO vector containing *IL-6* wild-type, mutant or *p63* wild-type or mutant 3′-UTR (0.1 μg) in Opti-MEM using Lipofectamine^®^ 3000 (Invitrogen). Cells were incubated for 48 h at 37 °C before firefly and Renilla luciferase activity was detected using a dual-luciferase reporter assay system (Promega) [40]. Firefly and Renilla luciferase activities were measured using a luminometer (SpectraMax iD5 Multi-Mode Microplate Reader; Molecular Devices). Luciferase activity was calculated as the ratio of firefly to Renilla luciferase activity and normalised to the empty dual-luciferase reporter vector and negative control for the mimic group [40]. Each assay was performed in triplicate and repeated three times.

### 2.8. Statistical Analysis

Data are expressed as mean ± standard error of the mean (SEM). One-way analysis of variance with the Bonferroni post-test was performed to compare group data. The unpaired *t*-test was used to compare two groups. *p* values of <0.05 were considered statistically significant. Statistical analysis was performed using GraphPad Prism Software (v8.0.3) [41]. Each experiment was repeated three or more times.

## 3. Results

### 3.1. miR-149-5p Expression in Airway Epithelial Cells after Poly (I:C) Incubation or S1 or S2 Transfection

**Figure 1:**
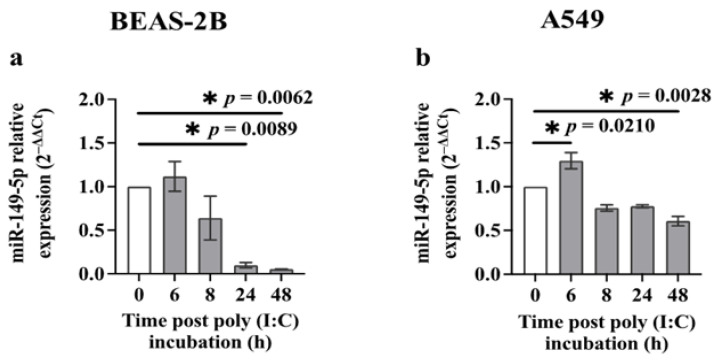
Poly (I:C) suppresses miR-149-5p expression in airway epithelial cells. BEAS-2B or A549 cells were cultured in a 24-well plate at 1 × 10^5^ cells per well in a BEGM or F-12K medium (FBS, 10%), respectively, for 24 h. Cells were then incubated in a BEBM or F-12K medium containing ITS+1 (1%) with poly (I:C) (0.5 μg/mL). miR-149-5p levels were assessed at 0, 6, 8, 24 and 48 h using RT-qPCR. (**a**) miR-149-5p expression in BEAS-2B cells after incubation with poly (I:C). (**b**) miR-149-5p expression in A549 cells after incubation with poly (I:C). The cycle threshold (Ct) value of miR-149-5p was normalised to that of RNU44 (ΔCt). Data are presented relative to the control at baseline (ΔΔCt) as mean ± SEM. * *p* ≤ 0.05, compared with the control group, using one-way analysis of variance with Bonferroni post-test, *n* = 3.

There are different reports on the concentrations of poly (I:C) used to stimulate epithelial cells [20,42,43]. Thus, we initially investigated the dose-dependent effect of poly (I:C) (0.01–10 μg/mL) on the viability of BEAS-2B or A549 cells. Microscopic images of BEAS-2B or A549 cell cultures showed that there were no significant changes in the morphology of cells incubated with or without poly (I:C) (10 μg/m) after 24 or 48 h (Figure S1a–h). Poly (I:C) at 0.5 μg/mL was not highly toxic in BEAS-2B cells and caused no cell death in A549 cells using the LDH assay [28] (Figure S1i–l). We then investigated the effect on miR-149-5p expression by incubating BEAS-2B cells or A549 cells with poly (I:C) (0.5 μg/mL) and assessing its expression at baseline and after 6, 8, 24 and 48 h using RT-qPCR. BEAS-2B cells expressed substantially lower levels of miR-149-5p after incubation with poly (I:C) for 24 (*p* = 0.0089) and 48 h (*p* = 0.0062) (Figure 1a). A549 cells incubated with poly (I:C) also exhibited significantly decreased expression of miR-149-5p levels after 48 h (*p* = 0.0028) (Figure 1b).

**Figure 2:**
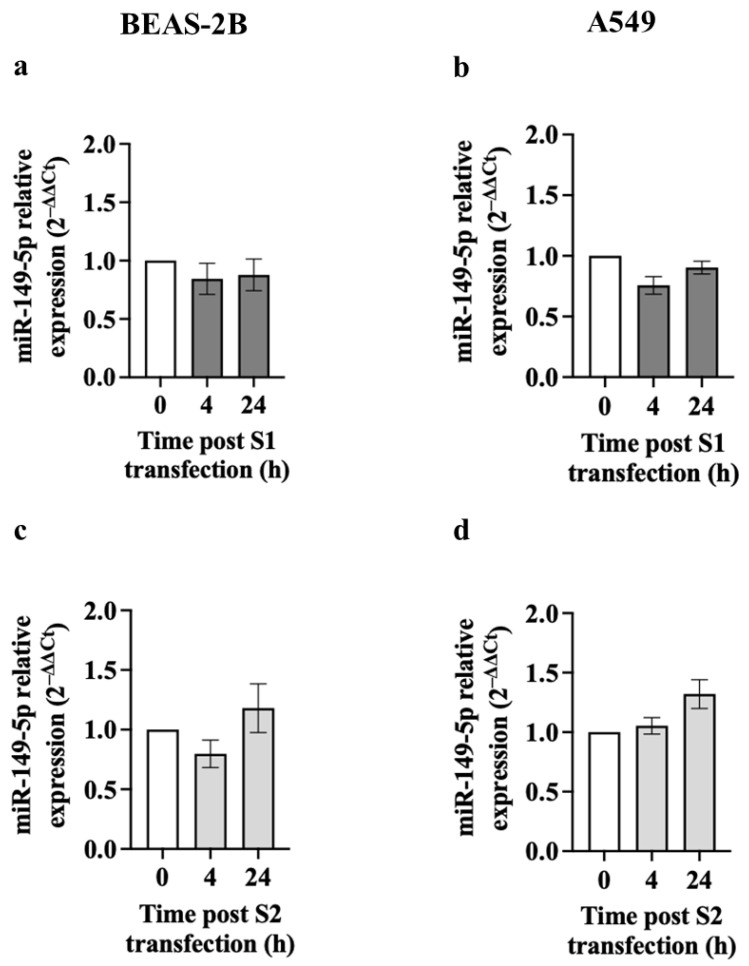
Effect of S1 or S2 transfection on miR-149-5p levels in airway epithelial cells. BEAS-2B or A549 cells were cultured in a 24-well plate at 1 × 10^5^ cells per well in a BEGM or F-12K medium (FBS, 10%), respectively, for 24 h. BEAS-2B or A549 cells were transfected with S1 or S2 (1 μg). Cells were harvested at baseline and after S1 or S2 transfection for 4 or 24 h. miR-149-5p expression was assessed using RT-qPCR. (**a**) miR-149-5p expression in BEAS-2B cells after transfection with S1, *n* = 5. (**b**) miR-149-5p expression in A549 cells after transfection with S1, *n* = 3. (**c**) miR-149-5p expression in BEAS-2B cells after transfection with S2, *n* = 5. (**d**) miR-149-5p expression in A549 cells after transfection with S2, *n* = 3. The cycle threshold (Ct) value of miR-149-5p was normalised to that of RNU44 (ΔCt). Data are presented relative to the control at baseline (ΔΔCt) as mean ± SEM.

Next, BEAS-2B and A549 cells were transfected with S1 or S2 (1 μg) for 4 or 24 h [38]. Initially, we transfected GFP (1 μg) into BEAS-2B and A549 cells for 4 h to confirm transfection efficiency by fluorescence microscopy [38]. Most GFP-positive cells exhibited evenly scattered GFP expression throughout the cells (Figure S2a–d). Furthermore, our results showed that transfection with the S1 or S2 for 24 h did not affect the morphology of BEAS-2B (Figure S2e–g) or A549 cells (Figure S2h–j). Moreover, neither S1 nor S2 transfection for 24 h affected the viability of BEAS-2B (Figure S2k) and A549 cells (Figure S2l). In contrast to poly (I:C), miR-149-5p expression is not significantly affected by transfection with S1 or S2 into BEAS-2B or A549 cells after 4 or 24 h compared to the baseline control (Figure 2). The transfection reagent (transfection control) at 4 or 24 h post-transfection did not affect miR-149-5p expressions compared with baseline (Figure S3) and hence was excluded from the data presented in Figure 2.

We next investigated the effect of poly (I:C), S1 or S2 on IL-6, a putative target of miR-149-5p [33,34].

### 3.2. IL-6 Release from Airway Epithelial Cells after Poly (I:C) Incubation or S1 or S2 Transfection

**Figure 3:**
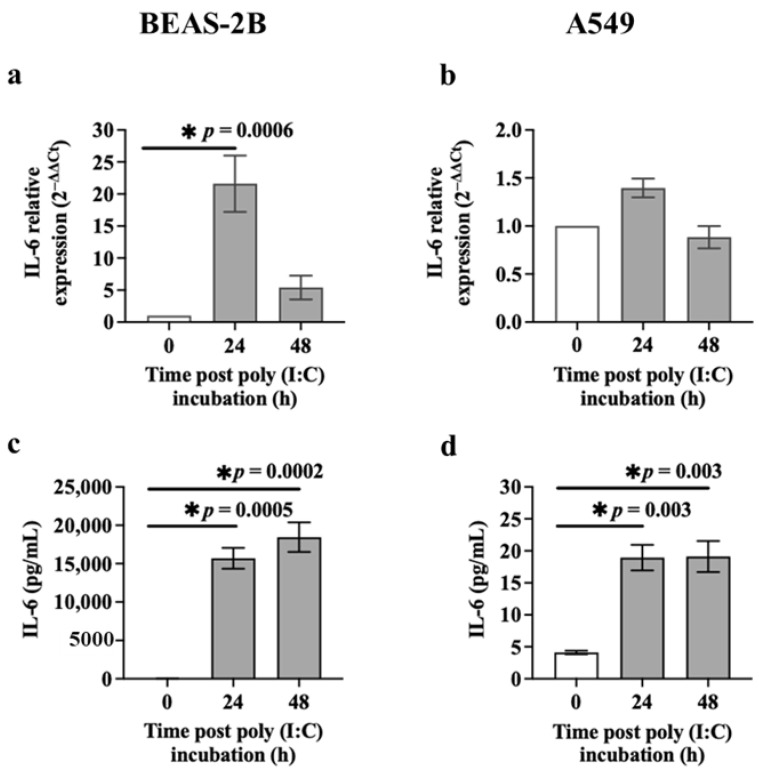
Effect of poly (I:C) on IL-6 expression in airway epithelial cells. BEAS-2B or A549 cells were cultured in a 24-well plate at 1 × 10^5^ cells/well in a BEGM or F-12K medium (FBS, 10%), respectively, for 24 h. BEAS-2B or A549 cells were incubated in a BEBM or F-12K medium containing ITS+1 (1%) with poly (I:C) (0.5 μg/mL). IL-6 mRNA and protein levels were assessed at baseline and after poly (I:C) incubation (24 or 48 h) using RT-qPCR and the ELISA technique, respectively. (**a**) The *IL-6* mRNA expression in BEAS-2B cells after poly (I:C) incubation, *n* = 5. (**b**) The *IL-6* mRNA expression in A549 cells after poly (I:C) incubation, *n* = 3. The Ct value of *IL-6* was normalised to that of 18S rRNA (ΔCt). Data are presented relative to the control at baseline (ΔΔCt). (**c**) Release of IL-6 from BEAS-2B cells after poly (I:C) incubation, *n* = 3. (**d**) Release of IL-6 from A549 cells after poly (I:C) incubation, *n* = 3. Data are presented as mean ± SEM. * *p* ≤ 0.05, compared with the control group, using one-way analysis of variance with Bonferroni post-test.

First, we assessed the *IL-6* mRNA expression in BEAS-2B and A549 cells exposed to poly (I:C) (0.5 μg/mL) using RT-qPCR. Poly (I:C)-challenged BEAS-2B cells expressed significantly higher levels of *IL-6* mRNA after 24 h (*p* = 0.0006) compared to baseline (Figure 3a). However, poly (I:C) incubation for 24 or 48 h did not affect *IL-6* mRNA expression in A549 cells compared to baseline (Figure 3b). We also assessed IL-6 release in the culture supernatant of cells incubated with poly (I:C) (0.5 μg/mL) for 24 or 48 h using ELISA. BEAS-2B cells incubated with poly (I:C) released substantially higher levels of IL-6 after 24 h (15,703.67 ± 1350.67 pg/mL (~900-fold greater than baseline, *p* = 0.0005)) or 48 h (18,453.00 ± 1927.29 pg/mL (~1000-fold greater than baseline, *p* = 0.0002)) compared to cells at baseline (18.33 ± 4.41 pg/mL) (mean ± SEM) (Figure 3c). A549 cells exposed to poly (I:C) over time also expressed higher levels of IL-6 (*p* = 0.003) at 24 h (18.94 ± 2.01 pg/mL) (~5-fold greater than baseline) or 48 h (19.12 ± 2.42 pg/mL) (~5-fold greater than baseline) compared to the baseline (4.12 ± 0.28 pg/mL) (Figure 3d). Interestingly, the expression of IL-6 at mRNA and protein levels were much greater in BEAS-2B compared to A549 cells incubated with poly (I:C) (Figure 3).

**Figure 4:**
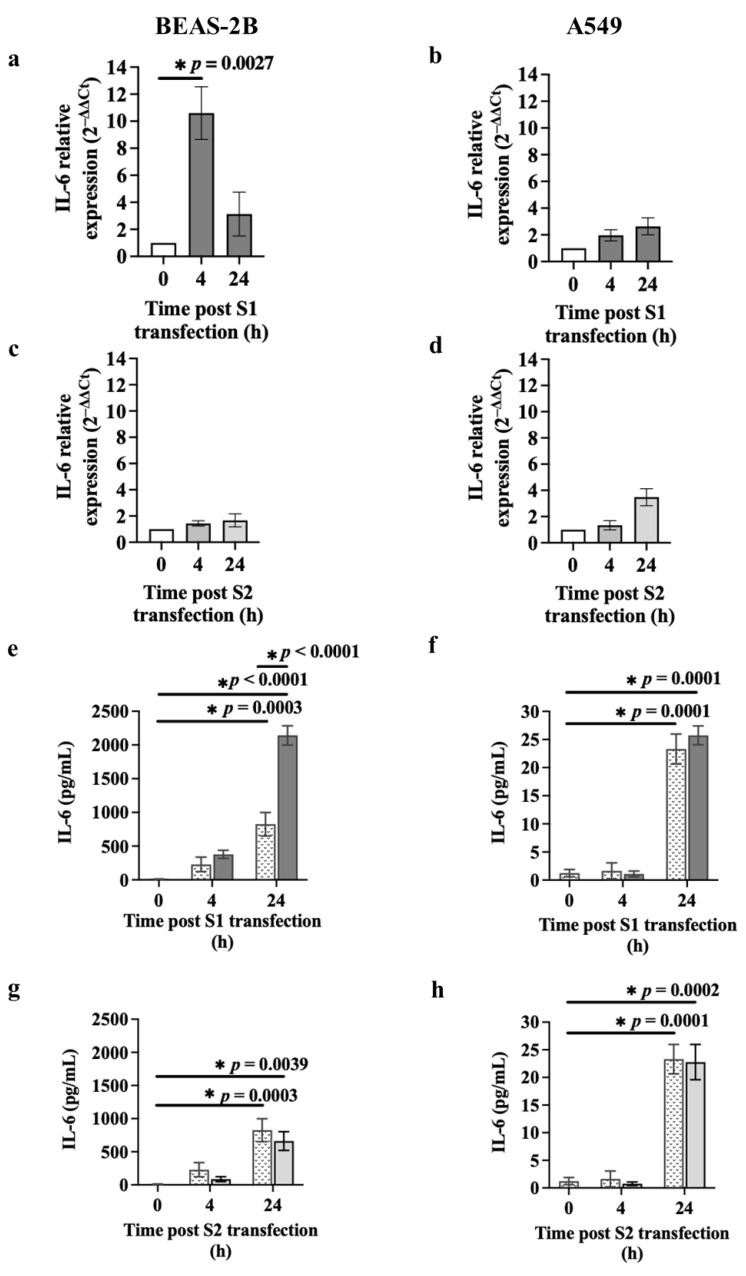
Effect of S1 or S2 on IL-6 expression in airway epithelial cells. BEAS-2B or A549 cells were cultured in a 24-well plate at 1 × 10^5^ cells per well in a BEGM or F-12K medium (FBS, 10%), respectively, for 24 h. BEAS-2B or A549 cells were transfected with S1 or S2 (1 μg). IL-6 mRNA and protein levels were assessed using the RT-qPCR and ELISA technique, respectively. (**a**) The *IL-6* mRNA expression after S1 transfection in BEAS-2B cells, *n* = 3. (**b**) The *IL-6* mRNA levels after S1 transfection in A549 cells, *n* = 3. (**c**) The *IL-6* mRNA levels after S2 transfection in BEAS-2B cells, *n* = 3. (**d**) The *IL-6* mRNA levels after S2 transfection in A549 cells, *n* = 3. The Ct value of *IL-6* was normalised to that of 18S rRNA (ΔCt). Data are presented relative to the control at baseline (ΔΔCt) as mean ± SEM. (**e**) IL-6 release after S1 transfection into BEAS-2B cells, *n* = 6. (**f**) IL-6 release after S1 transfection into A549 cells, *n* = 3. (**g**) IL-6 release after S2 transfection into BEAS-2B cells, *n* = 6. (**h**) IL-6 release after S2 transfection into A549 cells, *n* = 3. White, dotted, dark grey and light grey bars represent, baseline, transfection control, S1 transfection and S2 transfection, respectively. Data are presented as mean ± SEM. * *p* ≤ 0.05, compared with the control group, using one-way analysis of variance with Bonferroni post-test.

We also assessed the levels of IL-6 mRNA and protein in BEAS-2B cells and A549 cells transfected with S1 or S2 (1 μg) (Figure 4). There was no significant difference in *IL-6* expression between baseline and transfection control at 4 or 24 h (Figure S4); therefore, those data are not presented in Figure 4a–d. S1 transfection for 4 h upregulated *IL-6* mRNA expression compared to baseline (*p* = 0.0027) in BEAS-2B (Figure 4a) but not A549 cells (Figure 4b). Interestingly, S2 transfection had no effect on *IL-6* mRNA expression in BEAS-2B (Figure 4c) or A549 (Figure 4d) cells. S1 transfection substantially increased (~3-fold) IL-6 release (2141.83 ± 143.44 pg/mL) from BEAS-2B cells after 24 h compared to the transfection control (825.35 ± 173.41 pg/mL) (mean ± SEM) (*p* < 0.0001) (Figure 4e) but not in A549 cells (Figure 4f). In contrast, S2 transfection did not induce IL-6 release in BEAS-2B cells (Figure 4g) or A549 cells (Figure 4h) compared with the relative transfection control.

### 3.3. p63 Expression in Airway Epithelial Cells after Poly (I:C) Incubation or S1 or S2 Transfection

**Figure 5:**
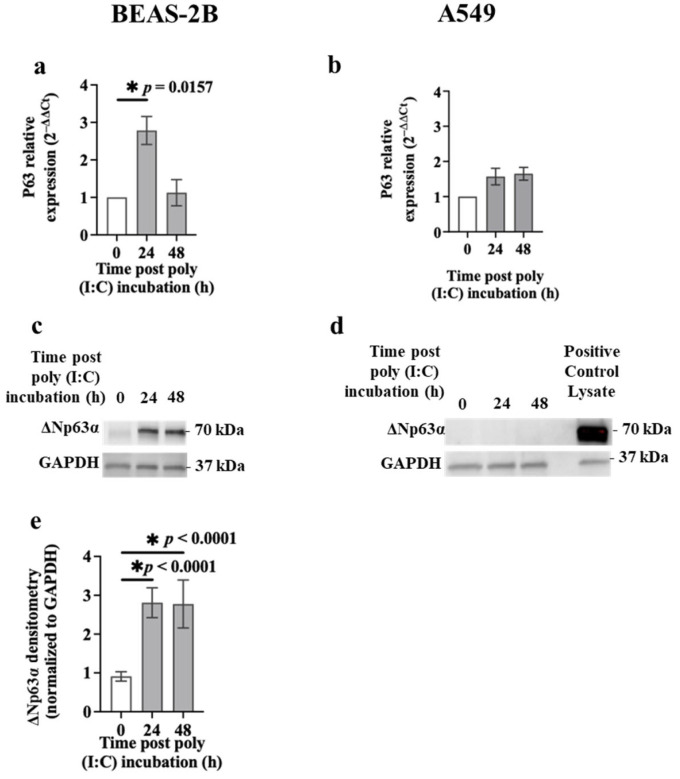
Effect of poly (I:C) challenge on p63 expression in airway epithelial cells. BEAS-2B or A549 cells were cultured in a 24-well plate at 1 × 10^5^ cells per well in a BEGM or F-12K medium (FBS, 10%), respectively, for 24 h. BEAS-2B or A549 cells were incubated in a BEBM or F-12K medium containing ITS+1 (1%) with poly (I:C) (0.5 μg/mL). *p63* mRNA and ΔNp63α protein expression was assessed at baseline and after poly (I:C) challenge (24 or 48 h) using RT-qPCR and immunoblotting, respectively. (**a**) *p63* mRNA expression in BEAS-2B cells, *n* = 3. (**b**) *p63* mRNA expression in A549 cells, *n* = 3. The Ct value of *p63* was normalised to that of 18S rRNA (ΔCt). Data are presented relative to the control at baseline (ΔΔCt). (**c**) Immunoblots representative of ΔNp63α protein in BEAS-2B cells, *n* = 5. (**d**) Immunoblots representative of ΔNp63α protein in A549 cells, *n* = 3. (**e**) Densitometric quantification of the immunoblot in BEAS-2B cells. Values were normalised to those of GAPDH (37 kDa) as a loading control. Data are presented as mean ± SEM. * *p* ≤ 0.05, compared with the control group, using one-way analysis of variance with Bonferroni post-test.

p63 is another potential target of miR-149-5p [33,34]. Therefore, we assessed the changes in *p63* mRNA and ΔNp63α protein expression in airway epithelial cells incubated with poly (I:C) (0.5 μg/mL) using RT-qPCR and immunoblotting, respectively. Exposure to poly (I:C) increased *p63* mRNA levels (~3-fold) in BEAS-2B cells after 24 h (*p* = 0.0157) compared to baseline (Figure 5a). However, poly (I:C) incubation had no effect on *p63* mRNA expression in A549 cells (Figure 5b). BEASE-2B cells incubated with poly (I:C) also expressed higher level of ΔNp63α protein after 24 (2.81 ± 0.39 relative band intensity, *p* = 0.0250) and 48 h (2.78 ± 0.62 relative band intensity, *p* = 0.0278) compared to baseline (0.91 ± 0.12 relative band intensity) (mean ± SEM) (Figure 5c,e). However, ΔNp63α protein expression was undetectable in A549 cells before and after the poly (I:C) challenge (Figure 5d).

**Figure 6:**
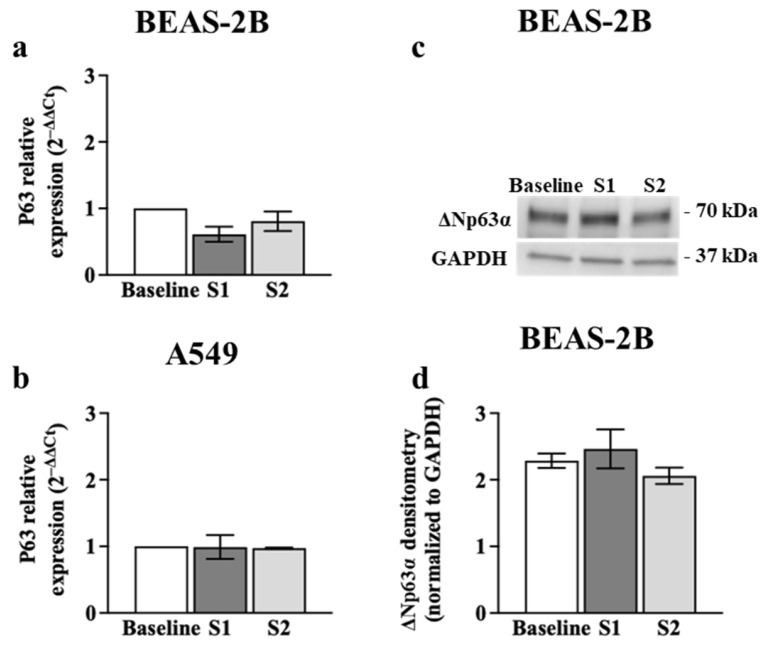
Effect of S1 or S2 transfection on p63 expression in airway epithelial cells. BEAS-2B or A549 cells were cultured in a 24-well plate at 1 × 10^5^ cells per well in a BEGM or F-12K medium (FBS, 10%), respectively, for 24 h. Cells were transfected with S1 or S2 (1 μg). *p63* mRNA and ΔNp63α protein levels were assessed using RT-qPCR and immunoblotting, respectively. (**a**) *p63* mRNA expression in BEAS-2B cells after transfection with S1 or S2 for 24 h, *n* = 5. (**b**) *p63* mRNA expression in A549 cells after transfection with S1 or S2 for 24 h, *n* = 3. The Ct value of *p63* mRNA was normalised to that of 18S rRNA (ΔCt). Data are presented relative to the control at baseline (ΔΔCt). (**c**) Immunoblot representative of ΔNp63α protein in BEAS-2B cells after transfection with S1 or S2 for 24 h, *n* = 5. (**d**) Densitometry analysis of the immunoblot. Values were normalised to those of GAPDH (37 kDa) as a loading control. Data are presented as mean ± SEM.

We also assessed the effect of the S1 or S2 (1 μg) on *p63* mRNA and ΔNp63α protein levels in airway epithelial cells. We showed that BEAS-2B and A549 cells transfected with S1 or S2 for 24 h expressed similar levels of *p63* mRNA (Figure 6a,b) compared to baseline. Similar patterns were detected in ΔNp63α protein levels after S1 or S2 transfection (Figure 6c,d) compared to baseline in BEAS-2B cells.

### 3.4. TLR3 Expression in Airway Epithelial Cells after Poly (I:C) Incubation

**Figure 7:**
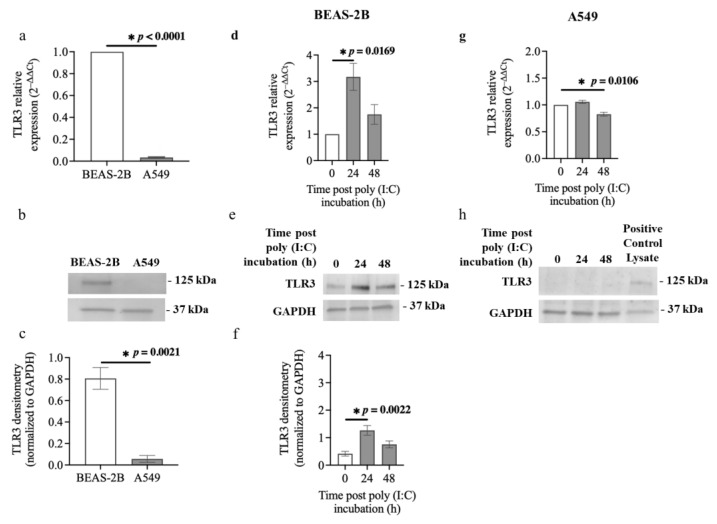
TLR3 expression in airway epithelial cells at baseline and after incubation with poly (I:C). BEAS-2B or A549 cells were cultured in a 24-well plate at 1 × 10^5^ cells per well in a BEGM or F-12K medium (FBS, 10%) for 24 h. BEAS-2B or A549 cells were incubated in a BEBM or F-12K medium containing ITS+1 (1%) with poly (I:C) (0.5 μg/mL) for 24 or 48 h. (**a**) *TLR3* mRNA expression at baseline in BEAS-2B and A549 cells, *n* = 3. The Ct value of *TLR3* was normalised to that of 18S rRNA (ΔCt). Data are presented relative to the BEAS-2B cells (ΔΔCt) as mean ± SEM. (**b**) Representative immunoblot and (**c**) densitometry analysis of TLR3 (125 kDa) at baseline in BEAS-2B cells and A549 cells, *n* = 3. GAPDH (37 kDa) was used as a loading control. Data are presented as mean ± SEM. Values were normalised to GAPDH as a loading control. (**d**) *TLR3* mRNA expression in BEAS-2B cells challenged with poly (I:C). The cycle threshold (Ct) value of TLR3 was normalised to that of 18S rRNA (ΔCt). Data are presented relative to the control at baseline (ΔΔCt), *n* = 3. (**e**) Representative immunoblot and (**f**) densitometry analysis of TLR3 in BEAS-2B cells incubated with poly (I:C), *n* = 5. (**g**) TLR3 mRNA expression in A549 cells challenged with poly (I:C), *n* = 3. (**h**) Representative immunoblot of TLR3 in A549 cells incubated with poly (I:C), *n* = 3. Data are presented as mean ± SEM. * *p* ≤ 0.05, compared with the control group, using unpaired *t*-test or one-way analysis of variance with Bonferroni post-test.

Poly (I:C) is recognised by TLR3 [13]. Our results indicate that BEAS-2B cells and A549 cells respond differently to poly (I:C) (Figure 3, Figure 5 and Figure S1). We therefore compared the mRNA and protein levels of TLR3 in BEAS-2B cells and A549 cells at baseline and after challenging with poly (I:C). A549 cells expressed substantially lower *TLR3* mRNA compared with BEAS-2B cells (*p* < 0.0001) at baseline (Figure 7a). Moreover, immunoblot results confirmed basal levels of TLR3 in BEAS-2B cells, but these were undetectable in A549 cells (Figure 7b,c). Furthermore, BEAS-2B cells challenged with poly (I:C) (0.5 μg/mL) for 24 h expressed significantly elevated *TLR3* mRNA (~3-fold) compared to the baseline control (*p* = 0.0169) (Figure 7d). *TLR3* mRNA expression increased slightly in response to poly (I:C) incubation after 48 h in BEAS-2B cells but did not reach statistical difference. Poly (I:C) challenge did substantially upregulate TLR3 protein levels (~3-fold) in BEAS-2B cells after 24 h (1.27 ± 0.17 relative band intensity) compared to the baseline control (0.41 ± 0.09 relative band intensity, *p* = 0.0022) (Figure 7e,f). In addition, the poly (I:C) challenge had no significant effect on *TLR3* mRNA expression for 24 h but suppressed *TLR3* mRNA expression for 48h compared to the baseline control in A549 cells (Figure 7g). TLR3 protein expression was too weak to be detected in A549 cells challenged with poly (I:C) for 24 or 48 h using the immunoblotting technique (Figure 7h).

### 3.5. TLR2 Expression in Airway Epithelial Cells after S1 or S2 Subunit Transfection

**Figure 8:**
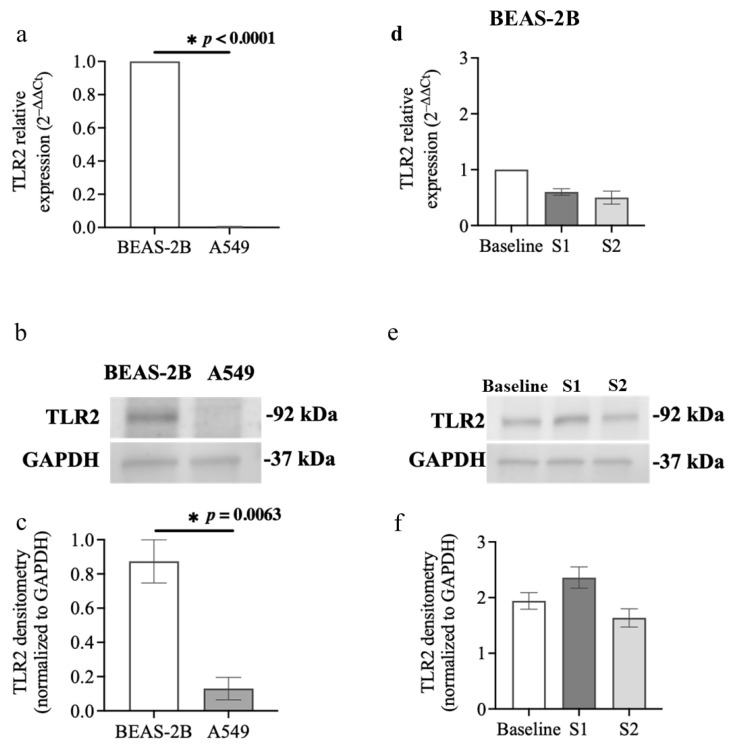
TLR2 expression in airway epithelial cells at baseline and after transfection with S1 or S2. BEAS-2B or A549 cells were cultured in a 24-well plate at 1 × 10^5^ cells per well in a BEGM or F-12K medium (FBS, 10%) for 24 h. TLR2 mRNA and protein levels were assessed using RT-qPCR and immunoblotting, respectively. (**a**) *TLR2* mRNA basal expression in BEAS-2B and A549 cells assessed, *n* = 3. Data are presented relative to control BEAS-2B cells (ΔΔCt) as mean ± SEM. (**b**) Representative immunoblot and (**c**) densitometry analysis of basal TLR2 protein expression (92 kDa) in BEAS-2B and A549 cells, *n* = 3. Values were normalised to those of GAPDH (37 kDa) as a loading control. (**d**) *TLR2* mRNA expression in BEAS-2B cells at baseline and after transfection with S1 or S2 subunit for 24 h, *n* = 3. The Ct value of *TLR2* mRNA was normalised to that of 18S rRNA (ΔCt). Data are presented relative to the control at baseline (ΔΔCt). (**e**) Representative immunoblot of TLR2 protein and (**f**) densitometry analysis in BEAS-2B cells at baseline and after transfection with S1 or S2 subunit for 24 h, *n* = 4. Values were normalised to those of GAPDH as a loading control. Data are presented as mean ± SEM. * *p* ≤ 0.05, compared with the control group, using unpaired *t*-test or one-way analysis of variance with Bonferroni post-test.

TLR2 has a significant role in inducing inflammatory responses during SARS-CoV-2 infection, and its expression is prominently increased in swab specimens of patients infected with SARS-CoV-2 [15,44]. Our results show that BEAS-2B and A549 cells respond differently to S1 and S2 transfection (Figure 4). We therefore compared the mRNA and protein expressions of TLR2 in BEAS-2B and A549 cells at basal level and after transfection with S1 or S2. Basal *TLR2* mRNA expression was significantly higher in BEAS-2B cells compared to A549 cells (*p* < 0.0001, Figure 8a). Consistent with mRNA expressions, BEAS-2B cells also had higher levels of TLR2 protein than A549 cells at basal level (Figure 8b,c). We also investigated whether S1 or S2 transfection could upregulate *TLR2* mRNA expression in airway epithelial cells. There was no significant difference in *TLR2* mRNA expression compared to baseline in BEAS-2B cells exposed to S1 or S2 for 24 h (Figure 8d). Further, TLR2 protein levels remained unchanged compared with baseline in BEAS-2B cells transfected with the S1 or S2 for 24 h (Figure 8e,f). *TLR2* mRNA was weakly expressed and almost undetectable in A549 cells at baseline and 24 h after transfection with S1 or S2 (Table S1).

### 3.6. Ectopic Expression of miR-149-5p Mimic Suppresses IL-6 and p63 Expression in BEAS-2B Cells

**Figure 9:**
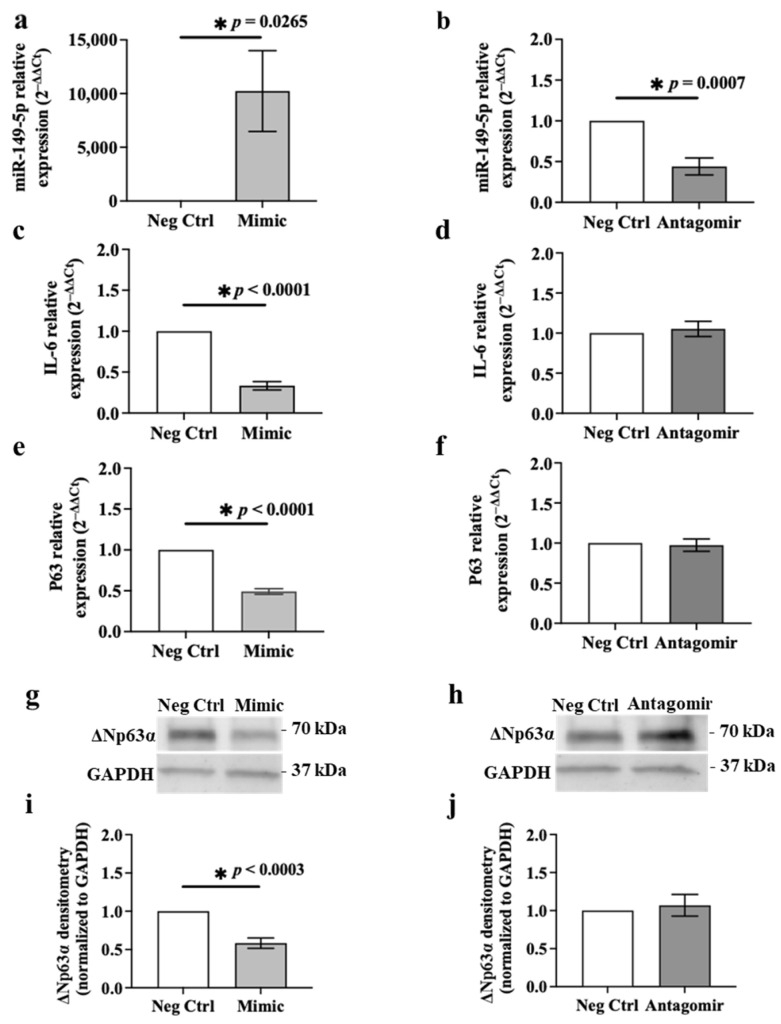
Effect of miR-149-5p mimic or antagomir transfection on miR-149-5p, IL-6 and p63 levels in BEAS-2B cells. BEAS-2B cells were cultured in a 24-well plate at 1 × 10^5^ cells/well in BEGM for 24 h. Cells were then transfected with the miR-149-5p mimic or antagomir (5 nM) in BEBM for 24 h (for miRNA and mRNA expressions) or 48 h (for protein levels). miR-149-5p expression in BEAS-2B cells after (**a**) miR-149-5p mimic transfection or (**b**) miR-149-5p antagomir transfection. The cycle threshold (Ct) value of miR-149-5p was normalised to that of RNU44 (ΔCt). Data are presented relative to the negative control (ΔΔCt) as mean ± SEM. *IL-6* mRNA expression in cells after transfection of (**c**) mimic or (**d**) antagomir. *p63* mRNA expression in cells after transfection of miR-149-5p (**e**) mimic or (**f**) antagomir. The Ct values of *IL-6* or *p63* mRNA was normalised to that of 18S rRNA (ΔCt). Data are presented relative to the negative control (ΔΔCt) as mean ± SEM. (**g**) Representative immunoblot and (**i**) densitometry analysis of ΔNp63α (70 kDa) protein in cells after transfection of miR-149-5p mimic. (**h**) Representative immunoblot and (**j**) densitometry analysis of ΔNp63α protein in cells after transfection of miR-149-5p antagomir. Values were normalised to those of GAPDH (37 kDa) as a loading control. Data are presented relative to the negative control as mean ± SEM. * *p* ≤ 0.05, compared to the control group, using an unpaired *t*-test, *n* = 5.

To confirm that IL-6 and p63 are targets of miR-149-5p, we transfected BEAS-2B cells with an miR-149-5p mimic or antagomir or the negative control for the mimic or antagomir (5 nM) for 24 or 48 h (Figure 9) [28]. Ectopic expression of the miR-149-5p mimic or antagomir (5 nM) did not affect cell viability compared to the relevant negative control using the LDH assay (Figure S5). Transfection of the miR-149-5p mimic or antagomir was confirmed by evaluating the miR-149-5p expression using RT-qPCR. Transfection with the miR-149-5p mimic significantly increased miR-149-5p expression in BEAS-2B compared to control cells after 24 h (*p* = 0.0265, Figure 9a). In contrast, miR-149-5p antagomir transfection suppressed miR-149-5p levels in BEAS-2B compared to control transfection after 24 h (*p* = 0.0007, Figure 9b). *IL-6* mRNA expression was downregulated in BEAS-2B transfected with the miR-149-5p mimic compared to the negative control after 24 h (*p* < 0.0001, Figure 9c). However, miR-149-5p antagomir transfection had no significant effect on *IL-6* mRNA expression in BEAS-2B cells after 24 h (Figure 9d). IL-6 protein levels in culture supernatants of BEAS-2B cells transfected with the miR-149-5p mimic or antagomir for 48 h were below the detection limits of the ELISA technique (Table S2). Transfection of BEAS-2B cells with the miR-149-5p mimic reduced *p63* mRNA expression compared to the control transfection after 24 h (*p* < 0.0001, Figure 9e). *p63* mRNA expression remained unchanged in BEAS-2B cells transfected with the miR-149-5p antagomir compared to the control transfection after 24 h (Figure 9f). In addition, over-expression of miR-149-5p suppressed ΔNp63α protein levels (0.58 ± 0.07 relative band intensity normalised to negative control) after 48 h (Figure 9g,i). miR-149-5p antagomir transfection had no effect on ΔNp63α protein expression in BEAS-2B cells after 48 h (Figure 9h,j).

### 3.7. Ectopic Expression of miR-149-5p Mimic Downregulates Poly (I:C)-Induced IL-6 Release in BEAS-2B Cells after 24 h

**Figure 10:**
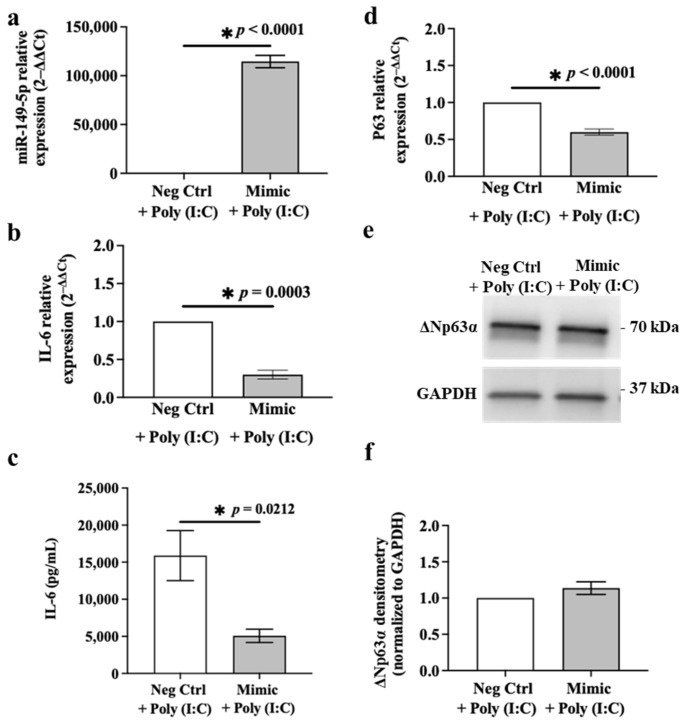
miR-149-5p mimic suppresses the poly (I:C)-induced IL-6 mRNA and protein and *p63* mRNA over-expression in BEAS-2B cells. BEAS-2B cells were cultured in a 24-well plate at 1 × 10^5^ cells per well in BEGM for 24 h. Cells were transfected with the miR-149-5p mimic (5 nM) in BEBM for 24 h and subsequently challenged with poly (I:C) (0.5 μg/mL) in BEBM (ITS+1, 1%) for 24 h. (**a**) miR-149-5p expression in BEAS-2B cells, *n* = 3. The Ct value of miR-149-5p was normalised to that of RNU44 (ΔCt). Data are presented relative to the negative control mimic (ΔΔCt). (**b**) *IL-6* mRNA expression in cells, *n* = 3. (**c**) IL-6 release from cells assessed using ELISA, *n* = 4. (**d**) *p63* mRNA expression in cells, *n* = 3. The Ct value of *IL-6* or *p63* mRNA was normalised to that of 18S rRNA (ΔCt). Data are presented relative to the negative control (ΔΔCt) as mean ± SEM. (**e**) Representative immunoblot and (**f**) densitometric quantification of ΔNp63α protein, *n* = 3. Values were normalised to those of GAPDH as a loading control. Data are presented relative to the negative control mimic as mean ± SEM. * *p* ≤ 0.05, compared with the control group, using an unpaired *t*-test.

miR-149-5p expression was supressed (Figure 1a), whereas mRNA and protein levels of IL-6 (Figure 3) and p63 (Figure 5) were increased, in BEAS-2B cells incubated with poly (I:C) (0.5 μg/mL) for 24 h. miR-149-5p mimic (5 nM) transfection for 24 h suppressed *IL-6* and *p63* mRNA levels in BEAS-2B cells (Figure 9a,c,e). To understand the potential of the miR-149-5p mimic in preventing or reversing poly (I:C) effects on IL-6 and/or p63, we transfected BEAS-2B cells with the miR-149-5p mimic (5 nM) for 24 h and subsequently incubated the cells with poly (I:C) (0.5 μg/mL) for 24 h. BEAS-2B cells transfected with miR-149-5p and then incubated with poly (I:C) expressed significantly higher levels of miR-149-5p (Figure 10a) but lower levels of IL-6 mRNA and protein (Figure 10b,c) compared with cells incubated with the negative control and poly (I:C) (*p* < 0.0001, *p* = 0.0003 and *p* = 0.0212, respectively). IL-6 levels released from BEAS-2B cells transfected with the negative control for the mimic or miR-149-5p mimic for 24 h and subsequently incubated with poly (I:C) for 24 h were 15,886.00 ± 3376.56 or 5068.50 ± 898.03 pg/mL (mean ± SEM), respectively. Although miR-149-5p mimic transfection and subsequent poly (I:C) incubation in BEAS-2B cells inhibited *p63* mRNA levels (*p* < 0.0001, Figure 10d), ΔNp63α protein remained unchanged compared to the negative control (Figure 10e,f).

### 3.8. Luciferase Reporter Assay

**Figure 11:**
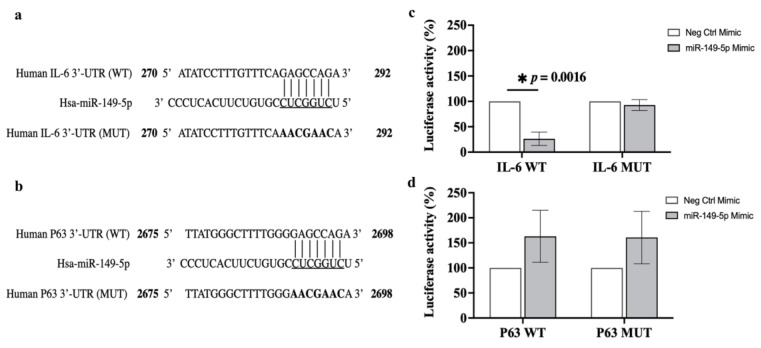
miR-149-5p targets the 3′-UTR of the mRNA of *IL-6*. (**a**) Representation of *IL-6* WT or MUT 3′-UTR position 270 to 292 from a total 3′-UTR length of 425 base pairs. Full-length *IL-6* WT or MUT 3′-UTR fragments were subcloned into a dual-luciferase reporter vector. (**b**) Representation of *p63* WT or MUT 3′-UTR position 2675 to 2698 from a total 3′-UTR length of 2774 base pairs. The *p63* WT or MUT 3′-UTR fragments (position 2357 to 2774) were subcloned into a dual-luciferase reporter vector. The underlined nucleotides are the putative miR-149-5p binding sites. Vertical black lines represent the potential miRNA binding site to the *IL-6* or *p63* WT 3′-UTR. Bold nucleotides are mutations generated in the *IL-6* or *p63* WT 3′-UTR by mutating the eight nucleotides potentially recognised by miR-149-5p. BEAS-2B cells were cultured in 96-well white clear-bottom plates at 4 × 10^4^ cells per well in BEGM and grown to 70% confluence. (**c**) Luciferase activity in BEAS-2B cells co-transfected with *IL-6* WT or MUT 3′-UTR vector (0.1 μg) and the mimic negative control (white bars) or miR-149-5p mimic (grey bars) (0.03 μM). (**d**) Luciferase activity in BEAS-2B cells co-transfected with *p63* WT or MUT 3′-UTR vector (0.1 μg) and the mimic negative control (white bars) or miR-149-5p mimic (grey bars) (0.03 μM). Luciferase activity was measured 48 h after transfection, calculated as the ratio of firefly to Renilla luciferase activity and normalised to the empty dual-luciferase reporter vector. Each assay was performed in triplicate. Data are presented relative to the mimic negative control as mean ± SEM, *n* = 3. * *p* ≤ 0.05, compared to the control, using one-way analysis of variance with Bonferroni post-test.

To confirm whether IL-6 or p63 is a direct target of miR-149-5p, a luciferase reporter assay was performed to detect the interaction between miR-149-5p and the 3′-UTR of *IL*-6 or *p63*. The potential binding sites of *IL-6* or *p63* wild-type (WT) or mutant (MUT) 3′-UTR were subcloned into a dual-luciferase reporter vector (Figure 11a,b). The construct was co-transfected into BEAS-2B cells along with the miR-149-5p mimic or negative control, and then luciferase activity was assessed. Co-transfection of the miR-149-5p mimic with *IL-6* WT resulted in a substantial decrease (~74%) in luciferase activity compared to the negative control (*p* = 0.0016, Figure 11c). There was no reduction in luciferase activity with co-transfection of the miR-149-5p mimic with *IL-6* MUT (Figure 11c). However, the luciferase reporter assay showed that co-transfection of the miR-149-5p mimic with *p63* WT or *p63* MUT had no significant effect on the luciferase activity compared to the negative control (Figure 11d). These results suggest that miR-149-5p directly targets the 3′-UTR of *IL-6* but not *p63*.

## 4. Discussion

miR-149-5p is located on chromosome 2 (Gene ID:406941) and is embedded in the first intron of the human glypican 1 (*GPC1*) gene [45]. miR-149-5p has emerged as a key regulator of cellular inflammatory responses and cell proliferation in different pathological conditions [31,46]. However, there is a gap in our knowledge about the function of miR-149-5p in airway epithelial cells during respiratory viral infection. Thus, we investigated the profile of miR-149-5p expression in airway epithelial cells exposed to poly (I:C), S1 or S2 as a model relevant to the pathophysiology of respiratory viruses. Our data revealed that poly (I:C) stimulation suppresses miR-149-5p expression, correlating with IL-6 and p63 upregulation and TLR3 overexpression in BEAS-2B cells. The response of A549 cells to poly (I:C) stimulation differed from BEAS-2B cells. Although A549 incubated with poly (I:C) expressed lower levels of miR-149-5p and higher levels of IL-6, the levels of p63 and TLR3 proteins were undetectable. Interestingly, miR-149-5p remained unchanged in both cell lines after exposure to S1 or S2, while S1 transfection induced IL-6 overexpression in BEAS-2B cells. Interestingly, BEAS-2B cells expressed TLR2, whereas A549 cells did not. However, S1 or S2 transfection had no effect on TLR2 expression in BEAS-2B cells. Ectopic over-expression of miR-149-5p in BEAS-2B cells suppressed IL-6 and p63 levels and inhibited poly (I:C)-induced IL-6 and p63. miR-149-5p directly suppressed IL-6 in BEAS-2B cells, as defined using the luciferase assay. Our data indicate that the distinct responses of miR-149-5p to respiratory viral models may differ in different types of airway epithelial cells. This may highlight potential specificity of miR-149-5p to TLR3-associated stimuli in specific cell types, with its potential as a therapeutic target to supress proinflammatory pathways.

We demonstrated that, upon challenge with poly (I:C) up to 48 h, miR-149-5p expression was substantially downregulated in BEAS-2B and A549 cells. These data for BEAS-2B cells are in agreement with previous reports by Hübner et al. [40], who showed that miR-149-5p directly targets chitinase-3-like-1, which is dependent on TNF-α-mediated NF-κB activation via IκBα phosphorylation [40]. Inflammation is a hallmark of severe respiratory viral infections in the lungs [47]. Respiratory epithelial cells respond to viral infections by generating pro-inflammatory cytokines and chemokines [12]. Accumulating evidence shows that IL-6 is released by airway epithelial cells and is associated with increased pulmonary inflammation and a cytokine storm [48,49]. We demonstrated that BEAS-2B cells challenged with poly (I:C) for 24 h expressed significantly elevated (~20-fold) *IL-6* mRNA levels compared to the baseline control. *IL-6* mRNA over-expression was correlated with increased IL-6 protein release by ~900-fold from BEAS-2B cells incubated with poly (I:C) for 24 h. Our data support the findings of Stowell et al., who reported significant cytokine secretion, including IL-6, from BEAS-2B cells exposed to poly (I:C) [20]. miRNA target prediction database tools predicted that the IL-6 gene may harbour a potential miRNA-binding site for miR-149-5p [33,34,35]. Further, we investigated whether poly (I:C)-induced IL-6 expression was post-transcriptionally regulated by miR-149-5p in BEAS-2B cells. We showed for the first time that ectopic over-expression of miR-149-5p for 24 h suppressed *IL-6* mRNA levels and inhibited poly (I:C)-induced IL-6 release in BEAS-2B cells. In addition, we demonstrated for the first time that miR-149-5p directly targets the 3′-UTR of *IL-6* in BEAS-2B cells. Interestingly, Li et al. reported that miR-149 also directly targets IL-6 in gastric stromal fibroblasts [32].

Previous evidence suggests that BEAS-2B cells incubated with poly (I:C) have reduced wound repair capacity [22]. The transcription factor p63 play an important role in epithelial wound repair [23]. Bioinformatics platforms suggest that p63 is a putative target of miR-149-5p [33,34,35]. Our data revealed that the downregulation of miR-149-5p was correlated with (~3-fold) upregulation of *p63* mRNA and ΔNp63α protein levels in BEAS-2B cells challenged with poly (I:C) for 24 h. We also found for the first time that ectopic over-expression of miR-149-5p in BEAS-2B cells downregulated *p63* mRNA and ΔNp63α protein levels after 24 and 48 h, respectively. miR-149-5p overexpression in BEASE-2B cells challenged with poly (I:C) also suppressed *p63* mRNA expression but did not affect its protein levels. However, a dual-luciferase assay revealed that miR-149-5p does not directly target p63. Interestingly, Sakaram et al. reported that p63 positively regulates miR-149-5p in a human keratinocyte cell line [50]. Our data and previous reports may indicate that cross-talk between TLR3-mediated miR-149-5p dysregulation and p63 may be indirect, complex and tissue/cell type specific.

Recent data have demonstrated that in patients with coronavirus disease (COVID-19), plasma levels of the SARS-CoV-2 spike protein S1 subunit correlate with disease progression [51]. Also, lung autopsy samples from patients with fatal COVID-19 showed alveolar-capillary barrier dysfunction, injury to alveolar and basal epithelial cells and defective tissue repair processes [52]. We observed that S1 or S2 had no effect on BEAS-2B and A549 cells viability, which is in agreement with data from Manfredelli et al. [18]. In contrast to the poly (I:C) model, miR-149-5p expression was not affected by S1 or S2 transfection in BEAS-2B or A549 cells. There was an increase in IL-6 secretion (~3-fold) in BEAS-2B cells exposed to S1 after 24h but not S2 or in A549 cells. Manfredelli et al. also reported that S1 induces IL-6 release from BEAS-2B cells [18]. In contrast to the poly (I:C) effect, we showed for the first time that S1 or S2 exposure does not affect p63 expression in BEAS-2B cells.

The different patterns of miR-149-5p expression in BEAS-2B cells challenged with poly (I:C), S1 or S2 indicates the specificity of miR-149-5p to different respiratory viral PAMPs. While TLR3 recognises poly (I:C), the expression of TLR2 is positively associated with the severity of SARS-CoV-2 infection [15,53]. Thus, the activation of specific TLRs in response to different PAMPs may contribute to the differential regulation of epigenetic mechanisms, including miR-149-5p, in airway epithelial cells. Our data suggest that miR-149-5p directly regulates the expression of IL-6 in BEAS-2B cells exposed to poly (I:C), whereas the proinflammatory role of S1 may be independent from miR-149-5p in these cells.

A549 cells responses to poly (I:C), S1 or S2 differed to those of BEAS-2B cells. After exposure to poly (I:C) (0.01–10 μg/mL) for 48 h, BEAS-2B cells had significant cell death, whereas the viability of A549 cells remained unaffected. The magnitude of IL-6 secretion from BEAS-2B cells challenged with poly (I:C) was ~1000-fold greater than a similar challenge in A549 cells. A549 cells did not express the ΔNp63α protein in the absence or presence of poly (I:C). Our data are consistent with previous reports of a lack of ΔNp63α in A549 cells at baseline [23,54]. Our data revealed that the S1 or S2 challenge had no effect on IL-6 expression in A549 cells. These are in contrast to the data of Patra et al., who showed that SARS-CoV-2 spike S1 protein in A549 cells promotes IL-6 release through an angiotensin II type 1 receptor (AT1)-mediated signalling cascade and induces the transcriptional regulatory molecules NF-κB and AP-1/c-Fos via MAPK activation [55]. While these authors used transient transfection with the SARS-CoV-2-Spike S1 or S2 region (Sino Biological) for ectopic expression of spike S1 or S2 in A549 cells [55], we transfected A549 cells with the S1 or S2 subunit protein (RayBiotech). In contrast to our data, Manfredelli et al. reported that the SARS-CoV-2 S1 spike protein induces IL-6 release in A549 cells [18]. While the source of A549 cells was similar, we cultured cells in an F-12K medium (recommended by ATCC), whereas Manfredelli et al. maintained cells in RPMI. Differences in the nature of the S1 or the media conditions may explain the different responses of A549 cells to S1 exposure.

We explored whether the different responses of BEAS-2B and A549 cells to poly (I:C) or S1 may be due to differences in the expression of TLRs that interact with these PAMPs and trigger cellular responses, including inflammation. Interestingly, TLR3 expression was significantly higher in BEAS-2B compared to A549 cells. Hillyer et al. also reported that BEAS-2B cells express higher *TRL3* mRNA levels than A549 cells [11]. Further, BEAS-2B cells challenged with poly (I:C) expressed higher levels of *TLR3* mRNA (~3-fold) and protein (~3-fold) compared to baseline. These results are in line with a previous report by Li et al. showing upregulation of *TLR3* mRNA in BEAS-2B cells incubated with poly (I:C) [56]. In contrast to BEAS-2B cells, A549 cells did not express TLR3 at baseline or after poly (I:C) challenge. Another study also showed that *TLR3* mRNA levels were undetectable in A549 cells and that a poly (I:C) challenge had no effect on TLR3 upregulation in A549 cells [57]. These data may explain the increased sensitivity of BEAS-2B to a poly (I:C) challenge, including cell death, higher IL-6 expression and p63 compared to A549 cells. Furthermore, BEAS-2B cells expressed significantly higher levels of TLR2 than A549 cells, which is aligned with the fundings of Hillyer et al. [11]. These data may support our observed increased sensitivity of BEAS-2B to S1 and hence elevated IL-6 in BEAS-2B but not A549 post S1 stimulation. Our data suggest that the difference in TLR3 and TLR2 expression between BEAS-2B and A549 cells may contribute to the different responses of cells to poly (I:C) and S1 or S2. This unique expression pattern of TLRs in BEAS-2B and A549 cells could be due to the source of these cell types. BEAS-2B cells were initiated from healthy human bronchial cells [36], whereas A549 cells were generated from a human alveolar cell carcinoma that belonged to different parts of the bronchial tree [58].

Our study has some limitations. We investigated the potential role of miR-149-5p in immortalised BEAS-2B and A549 cell lines. A549 cells are a model of the alveolar type II pulmonary epithelium but are neoplastic cells, which may affect the results. Further investigations in primary epithelial cells will increase the translational relevance of our findings. Moreover, BEAS-2B cells were maintained in submerged cultures that only represent basal epithelial cells. Our model did not provide a clear picture of the pseudostratified structure of bronchial epithelial cells. Thus, it will be interesting to obtain evidence of miR-149-5p function in epithelial cells differentiated in air–liquid interface conditions. Another limitation is that we did not perform dose-response studies on S1 or S2 transfection in airway epithelial cells. Future assessments of the response of these cells to multiple doses of the S1 or S2 may generate valuable outcomes. Finally, we used two respiratory virus PAMPs to determine the role of miR-149-5p in airway epithelial cells. Extending our studies to live respiratory viruses would be beneficial for validating the biological function of miR-149-5p during infection in airway epithelial cells. Expanding this investigation to the regulatory functions of miR-149-5p on other inflammatory cytokines and epithelial cell integrity markers will add value to this research. For more physiologically relevant models, expanding this research to co-culturing epithelial cells with macrophages will add value to investigating inflammatory and wound healing responses after viral challenges. Future verification of these data using in vivo models, for example, mice challenged with live viruses, will be essential for confirmation outcomes.

## 5. Conclusions

BEAS-2B cells respond differently to PAMPs of different respiratory viral infections, including poly (I:C), S1 or S2, compared to A549 cells. miR-149-5p directly targets IL-6 in BEAS-2B cells. TLR3-mediated miR-149-5p dysregulation may directly induce increased IL-6 and indirectly increase p63 expression in BEAS-2B cells. Our data showed for the first time that ectopic expression of miR-149-5p can suppress IL-6 and p63 expression and reverse poly (I:C)-induced IL-6 release in BEAS-2B cells. These findings support the potential role of miR-149-5p in modulating TLR3-mediated inflammatory responses in bronchial epithelial cells, which may inform future targeted approaches to therapy.

## Data Availability

All data generated or analysed during this study are included in this published article and are available from the corresponding author on request.

## Supplementary Materials

The following supporting information can be downloaded at: https://www.mdpi.com/article/10.3390/cells13110919/s1, Figure S1: Viability of airway epithelial cells incubated with different concentrations of poly (I:C); Figure S2: Effect of S1 or S2 transfection on viability of airway epithelial cells; Figure S3: Effect of transfection reagent on miR-149-5p levels in airway epithelial cells; Figure S4: Effect of transfection reagent on IL-6 mRNA levels in airway epithelial cells; Table S1: *TLR2* mRNA expression in A549 cell after S1 or S2 transfection; Figure S5: Effect of miR-149-5p mimic or antagomir transfection on the viability of BEAS-2B cells; Table S2: Effect of miR-149-5p mimic or antagomir transfection on the release of IL-6 in BEAS-2B cells.

## References

[1] Shi T., McLean K., Campbell H., Nair H. (2015). Aetiological role of common respiratory viruses in acute lower respiratory infections in children under five years: A systematic review and meta-analysis. J. Glob. Health.

[2] Mishra P., Nayak L., Das R.R., Dwibedi B., Singh A. (2016). Viral Agents Causing Acute Respiratory Infections in Children under Five: A Study from Eastern India. Int. J. Pediatr..

[3] Pollard C.A., Morran M.P., Nestor-Kalinoski A.L. (2020). The COVID-19 pandemic: A global health crisis. Physiol. Genom..

[4] Sullivan S.J., Jacobson R.M., Dowdle W.R., Poland G.A. (2010). 2009 H1N1 influenza. Mayo Clin. Proc..

[5] Johansen M.D., Mahbub R.M., Idrees S., Nguyen D.H., Miemczyk S., Pathinayake P., Nichol K., Hansbro N.G., Gearing L.J., Hertzog P.J. (2022). Increased SARS-CoV-2 Infection, Protease, and Inflammatory Responses in Chronic Obstructive Pulmonary Disease Primary Bronchial Epithelial Cells Defined with Single-Cell RNA Sequencing. Am. J. Respir. Crit. Care Med..

[6] Hsu A.C., Starkey M.R., Hanish I., Parsons K., Haw T.J., Howland L.J., Barr I., Mahony J.B., Foster P.S., Knight D.A. (2015). Targeting PI3K-p110alpha Suppresses Influenza Virus Infection in Chronic Obstructive Pulmonary Disease. Am. J. Respir. Crit. Care Med..

[7] Tam A., Wadsworth S., Dorscheid D., Man S.F., Sin D.D. (2011). The airway epithelium: More than just a structural barrier. Ther. Adv. Respir. Dis..

[8] Chan R.W., Yuen K.M., Yu W.C., Ho C.C., Nicholls J.M., Peiris J.S., Chan M.C. (2010). Influenza H5N1 and H1N1 virus replication and innate immune responses in bronchial epithelial cells are influenced by the state of differentiation. PLoS ONE.

[9] Lukassen S., Chua R.L., Trefzer T., Kahn N.C., Schneider M.A., Muley T., Winter H., Meister M., Veith C., Boots A.W. (2020). SARS-CoV-2 receptor ACE2 and TMPRSS2 are primarily expressed in bronchial transient secretory cells. Embo J..

[10] Yoshikawa T., Hill T.E., Yoshikawa N., Popov V.L., Galindo C.L., Garner H.R., Peters C.J., Tseng C.T. (2010). Dynamic innate immune responses of human bronchial epithelial cells to severe acute respiratory syndrome-associated coronavirus infection. PLoS ONE.

[11] Hillyer P., Shepard R., Uehling M., Krenz M., Sheikh F., Thayer K.R., Huang L., Yan L., Panda D., Luongo C. (2018). Differential Responses by Human Respiratory Epithelial Cell Lines to Respiratory Syncytial Virus Reflect Distinct Patterns of Infection Control. J. Virol..

[12] Vareille M., Kieninger E., Edwards M.R., Regamey N. (2011). The airway epithelium: Soldier in the fight against respiratory viruses. Clin. Microbiol. Rev..

[13] Mogensen T.H. (2009). Pathogen recognition and inflammatory signaling in innate immune defenses. Clin. Microbiol. Rev..

[14] Alexopoulou L., Holt A.C., Medzhitov R., Flavell R.A. (2001). Recognition of double-stranded RNA and activation of NF-kappaB by Toll-like receptor 3. Nature.

[15] Dai J., Wang Y., Wang H., Gao Z., Wang Y., Fang M., Shi S., Zhang P., Wang H., Su Y. (2022). Toll-Like Receptor Signaling in Severe Acute Respiratory Syndrome Coronavirus 2-Induced Innate Immune Responses and the Potential Application Value of Toll-Like Receptor Immunomodulators in Patients With Coronavirus Disease 2019. Front. Microbiol..

[16] Forsyth C.B., Zhang L., Bhushan A., Swanson B., Zhang L., Mamede J.I., Voigt R.M., Shaikh M., Engen P.A., Keshavarzian A. (2022). The SARS-CoV-2 S1 Spike Protein Promotes MAPK and NF-kB Activation in Human Lung Cells and Inflammatory Cytokine Production in Human Lung and Intestinal Epithelial Cells. Microorganisms.

[17] Khan S., Shafiei M.S., Longoria C., Schoggins J., Savani R.C., Zaki H. (2021). SARS-CoV-2 spike protein induces inflammation via TLR2-dependent activation of the NF-κB pathway. bioRxiv.

[18] Manfredelli D., Pariano M., Costantini C., Graziani A., Bozza S., Romani L., Puccetti P., Talesa V.N., Antognelli C. (2023). Severe Acute Respiratory Syndrome Coronavirus 2 (SARS-CoV-2) Spike Protein S1 Induces Methylglyoxal-Derived Hydroimidazolone/Receptor for Advanced Glycation End Products (MG-H1/RAGE) Activation to Promote Inflammation in Human Bronchial BEAS-2B Cells. Int. J. Mol. Sci..

[19] Cai J., Ma W., Wang X., Chang H., Wei Z., Li J., Zeng M. (2023). The spike protein of SARS-CoV-2 induces inflammation and EMT of lung epithelial cells and fibroblasts through the upregulation of GADD45A. Open Med. (Wars).

[20] Stowell N.C., Seideman J., Raymond H.A., Smalley K.A., Lamb R.J., Egenolf D.D., Bugelski P.J., Murray L.A., Marsters P.A., Bunting R.A. (2009). Long-term activation of TLR3 by Poly(I:C) induces inflammation and impairs lung function in mice. Respir. Res..

[21] Rincon M., Irvin C.G. (2012). Role of IL-6 in asthma and other inflammatory pulmonary diseases. Int. J. Biol. Sci..

[22] Lewandowska-Polak A., Brauncajs M., Jarzębska M., Pawełczyk M., Kurowski M., Chałubiński M., Makowska J., Kowalski M.L. (2018). Toll-Like Receptor Agonists Modulate Wound Regeneration in Airway Epithelial Cells. Int. J. Mol. Sci..

[23] Warner S.M., Hackett T.L., Shaheen F., Hallstrand T.S., Kicic A., Stick S.M., Knight D.A. (2013). Transcription factor p63 regulates key genes and wound repair in human airway epithelial basal cells. Am. J. Respir. Cell Mol. Biol..

[24] Cai Y., Yu X., Hu S., Yu J. (2009). A brief review on the mechanisms of miRNA regulation. Genom. Proteom. Bioinform..

[25] Foster P.S., Plank M., Collison A., Tay H.L., Kaiko G.E., Li J., Johnston S.L., Hansbro P.M., Kumar R.K., Yang M. (2013). The emerging role of microRNAs in regulating immune and inflammatory responses in the lung. Immunol. Rev..

[26] Moheimani F., Hsu A.C., Reid A.T., Williams T., Kicic A., Stick S.M., Hansbro P.M., Wark P.A., Knight D.A. (2016). The genetic and epigenetic landscapes of the epithelium in asthma. Respir. Res..

[27] Lam W.Y., Yeung A.C., Ngai K.L., Li M.S., To K.F., Tsui S.K., Chan P.K. (2013). Effect of avian influenza A H5N1 infection on the expression of microRNA-141 in human respiratory epithelial cells. BMC Microbiol..

[28] Moheimani F., Koops J., Williams T., Reid A.T., Hansbro P.M., Wark P.A., Knight D.A. (2018). Influenza A virus infection dysregulates the expression of microRNA-22 and its targets; CD147 and HDAC4, in epithelium of asthmatics. Respir. Res..

[29] Tahamtan A., Inchley C.S., Marzban M., Tavakoli-Yaraki M., Teymoori-Rad M., Nakstad B., Salimi V. (2016). The role of microRNAs in respiratory viral infection: Friend or foe?. Rev. Med. Virol..

[30] Zhi Y., Zhou H., Mubalake A., Chen Y., Zhang B., Zhang K., Chu X., Wang R. (2018). Regulation and functions of MicroRNA-149 in human cancers. Cell Prolif..

[31] Ren F.-J., Yao Y., Cai X.-Y., Cai Y.-T., Su Q., Fang G.-Y. (2021). MiR-149-5p: An Important miRNA Regulated by Competing Endogenous RNAs in Diverse Human Cancers. Front. Oncol..

[32] Li P., Shan J.-X., Chen X.-H., Zhang D., Su L.-P., Huang X.-Y., Yu B.-Q., Zhi Q.-M., Li C.-L., Wang Y.-Q. (2015). Epigenetic silencing of microRNA-149 in cancer-associated fibroblasts mediates prostaglandin E2/interleukin-6 signaling in the tumor microenvironment. Cell Res..

[33] Targetscan. https://www.targetscan.org/cgi-bin/targetscan/vert_72/targetscan.cgi?species=Human&gid=&mir_sc=&mir_c=&mir_nc=&mir_vnc=&mirg=miR-149-5p.

[34] miRDB. http://www.mirdb.org/.

[35] Rennie W., Liu C., Carmack C.S., Wolenc A., Kanoria S., Lu J., Long D., Ding Y. (2014). STarMir: A web server for prediction of microRNA binding sites. Nucleic Acids Res..

[36] Reddel R.R., Ke Y., Gerwin B.I., McMenamin M.G., Lechner J.F., Su R.T., Brash D.E., Park J.B., Rhim J.S., Harris C.C. (1988). Transformation of human bronchial epithelial cells by infection with SV40 or adenovirus-12 SV40 hybrid virus, or transfection via strontium phosphate coprecipitation with a plasmid containing SV40 early region genes. Cancer Res..

[37] Foster K.A., Oster C.G., Mayer M.M., Avery M.L., Audus K.L. (1998). Characterization of the A549 cell line as a type II pulmonary epithelial cell model for drug metabolism. Exp. Cell Res..

[38] Bortolotti D., Gentili V., Rizzo S., Rotola A., Rizzo R. (2020). SARS-CoV-2 Spike 1 Protein Controls Natural Killer Cell Activation via the HLA-E/NKG2A Pathway. Cells.

[39] Image Lab Software https://www.bio-rad.com/en-uk/product/image-lab-software?ID=KRE6P5E8Z.

[40] Hübner K., Karwelat D., Pietsch E., Beinborn I., Winterberg S., Bedenbender K., Benedikter B.J., Schmeck B., Vollmeister E. (2020). NF-κB-mediated inhibition of microRNA-149-5p regulates Chitinase-3-like 1 expression in human airway epithelial cells. Cell. Signal..

[41] Dotmatics. https://www.graphpad.com/.

[42] Koizumi Y., Nagase H., Nakajima T., Kawamura M., Ohta K. (2016). Toll-like receptor 3 ligand specifically induced bronchial epithelial cell death in caspase dependent manner and functionally upregulated Fas expression. Allergol. Int..

[43] Sada M., Watanabe M., Inui T., Nakamoto K., Hirata A., Nakamura M., Honda K., Saraya T., Kurai D., Kimura H. (2021). Ruxolitinib inhibits poly(I:C) and type 2 cytokines-induced CCL5 production in bronchial epithelial cells: A potential therapeutic agent for severe eosinophilic asthma. Immun. Inflamm. Dis..

[44] Mohanty M.C., Varose S.Y., Sawant U.P., Fernandes M.M. (2021). Expression of innate immune response genes in upper airway samples of SARS-CoV-2 infected patients: A preliminary study. Indian J. Med. Res..

[45] Jin L., Hu W.L., Jiang C.C., Wang J.X., Han C.C., Chu P., Zhang L.J., Thorne R.F., Wilmott J., Scolyer R.A. (2011). MicroRNA-149*, a p53-responsive microRNA, functions as an oncogenic regulator in human melanoma. Proc. Natl. Acad. Sci. USA.

[46] He Y., Yu D., Zhu L., Zhong S., Zhao J., Tang J. (2018). miR-149 in Human Cancer: A Systemic Review. J. Cancer.

[47] Christie M.J., Irving A.T., Forster S.C., Marsland B.J., Hansbro P.M., Hertzog P.J., Nold-Petry C.A., Nold M.F. (2021). Of bats and men: Immunomodulatory treatment options for COVID-19 guided by the immunopathology of SARS-CoV-2 infection. Sci. Immunol..

[48] Conti P., Ronconi G., Caraffa A., Gallenga C.E., Ross R., Frydas I., Kritas S.K. (2020). Induction of pro-inflammatory cytokines (IL-1 and IL-6) and lung inflammation by Coronavirus-19 (COVI-19 or SARS-CoV-2): Anti-inflammatory strategies. J. Biol. Regul. Homeost Agents.

[49] Wang X., Tang G., Liu Y., Zhang L., Chen B., Han Y., Fu Z., Wang L., Hu G., Ma Q. (2022). The role of IL-6 in coronavirus, especially in COVID-19. Front. Pharmacol..

[50] Sakaram S., Craig M.P., Hill N.T., Aljagthmi A., Garrido C., Paliy O., Bottomley M., Raymer M., Kadakia M.P. (2018). Identification of novel ΔNp63α-regulated miRNAs using an optimized small RNA-Seq analysis pipeline. Sci. Rep..

[51] Ogata A.F., Maley A.M., Wu C., Gilboa T., Norman M., Lazarovits R., Mao C.-P., Newton G., Chang M., Nguyen K. (2020). Ultra-Sensitive Serial Profiling of SARS-CoV-2 Antigens and Antibodies in Plasma to Understand Disease Progression in COVID-19 Patients with Severe Disease. Clin. Chem..

[52] D’Agnillo F., Walters K.A., Xiao Y., Sheng Z.M., Scherler K., Park J., Gygli S., Rosas L.A., Sadtler K., Kalish H. (2021). Lung epithelial and endothelial damage, loss of tissue repair, inhibition of fibrinolysis, and cellular senescence in fatal COVID-19. Sci. Transl. Med..

[53] Zheng M., Karki R., Williams E.P., Yang D., Fitzpatrick E., Vogel P., Jonsson C.B., Kanneganti T.D. (2021). TLR2 senses the SARS-CoV-2 envelope protein to produce inflammatory cytokines. Nat. Immunol..

[54] Xu J., Li F., Gao Y., Guo R., Ding L., Fu M., Yi Y., Chen H., Xiao Z.J., Niu M. (2021). E47 upregulates DeltaNp63alpha to promote growth of squamous cell carcinoma. Cell Death Dis..

[55] Patra T., Meyer K., Geerling L., Isbell T.S., Hoft D.F., Brien J., Pinto A.K., Ray R.B., Ray R. (2020). SARS-CoV-2 spike protein promotes IL-6 trans-signaling by activation of angiotensin II receptor signaling in epithelial cells. PLoS Pathog..

[56] Li Y.G., Siripanyaphinyo U., Tumkosit U., Noranate N., A A.N., Pan Y., Kameoka M., Kurosu T., Ikuta K., Takeda N. (2012). Poly (I:C), an agonist of toll-like receptor-3, inhibits replication of the Chikungunya virus in BEAS-2B cells. Virol. J..

[57] Tissari J., Sirén J., Meri S., Julkunen I., Matikainen S. (2005). IFN-α Enhances TLR3-Mediated Antiviral Cytokine Expression in Human Endothelial and Epithelial Cells by Up-Regulating TLR3 Expression1. J. Immunol..

[58] Lieber M., Smith B., Szakal A., Nelson-Rees W., Todaro G. (1976). A continuous tumor-cell line from a human lung carcinoma with properties of type II alveolar epithelial cells. Int. J. Cancer.

