# Supplementary material for: Distinct Effects of Respiratory Viral Infection Models on miR-149-5p, IL-6 and p63 Expression in BEAS-2B and A549 Epithelial Cells
Source: Cells. 2024 May 26;13(11):919. doi: 10.3390/cells13110919 (PMC11172188; doi:10.3390/cells13110919)
Supplement: Supplementary file 1 [file cells-13-00919-s001.zip › cells-3009592-supplementary.pdf]

Title: Distinct effects of respiratory viral infection models on miR-149-5p, IL-6 and p63 expression in BEAS-2B and A549 epithelial cells

Authors: Nafeesa Shahdab[1], Christopher Ward[2], Philip M. Hansbro[3], Stephen Cummings[1], John S. Young[1], Fatemeh Moheimani[4,*]

Affiliations:

1 National Horizons Centre, School of Health and Life Sciences, Teesside University, Middlesbrough, UK; N.Shahdab@tees.ac.uk, S.Cummings@tees.ac.uk, J.Young@tees.ac.uk

2 Translational and Clinical Research Institute, Newcastle University, Newcastle upon Tyne, UK; chris.ward@newcastle.ac.uk

3 Centre for Inflammation, Centenary Institute and University of Technology Sydney, Faculty of Science, School of Life Sciences, Sydney, Australia; Philip.Hansbro@uts.edu.au

4 Department of Life Sciences, Manchester Metropolitan University, Manchester, UK; f.moheimani@mmu.ac.uk

* Correspondence: f.moheimani@mmu.ac.uk; Tel.: (+44)161 247 1189

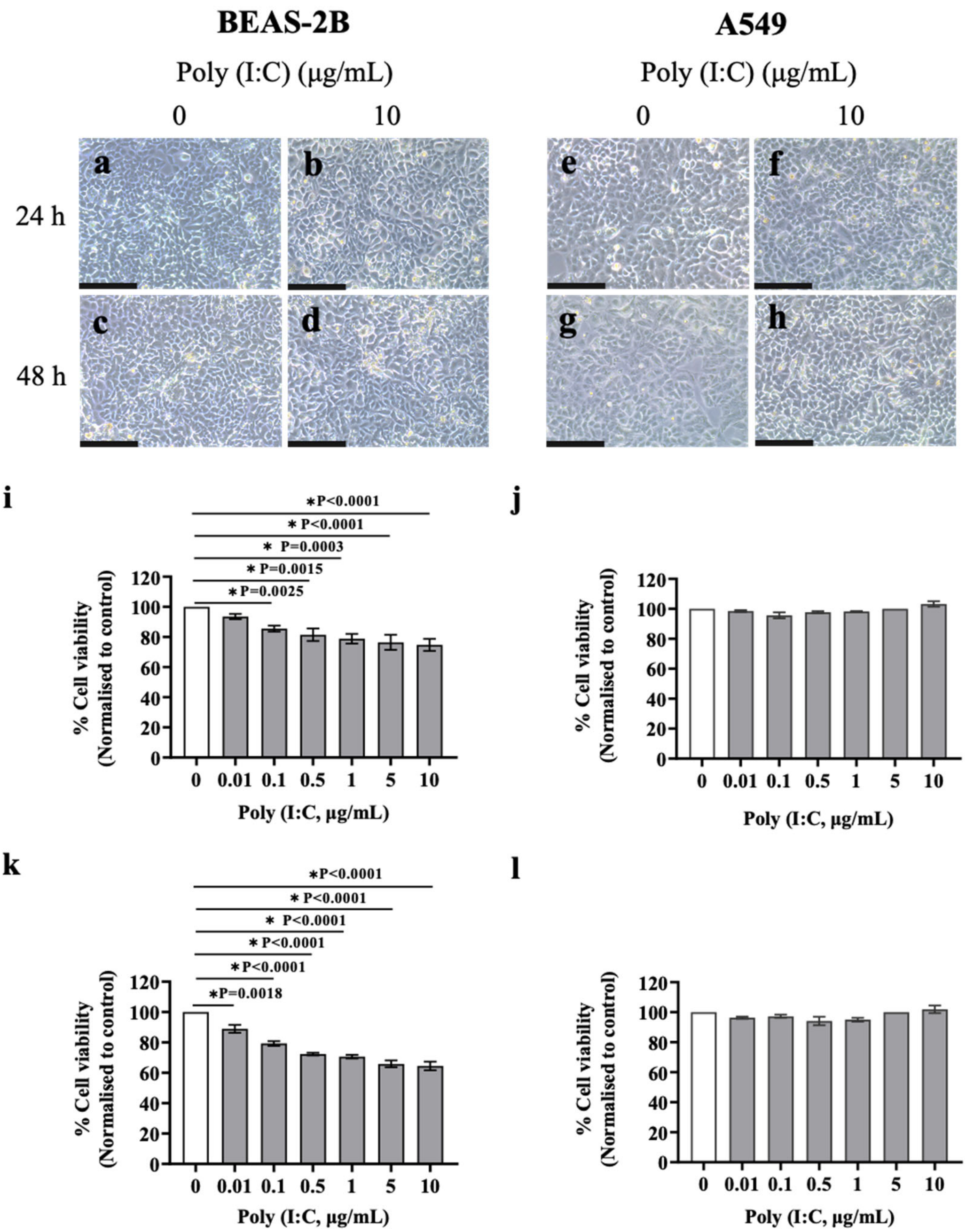

Figure S1. Viability of airway epithelial cells incubated with different concentrations of poly (I:C). BEAS-2B or A549 cells were incubated in BEBM or F-12K medium containing ITS+1 (1%) with poly (I:C) (0.01 - 10 µg/ml). (a) Microscopic images of control BEAS-2B cells incubated at 24 h. (b) Microscopic images of BEAS-2B cells incubated with poly (I:C) (10 µg/ml) for 24 h. (c) Microscopic images of control BEAS-2B cells incubated at 48 h. (d) Microscopic images of BEAS-2B cells incubated with poly (I:C) (10 µg/ml) for 48 h. (e) Microscopic images of control A549 cells at 24 h. (f) Microscopic images of A549 cells incubated with poly (I:C) (10 µg/ml) for 24 h. (g) Microscopic images of control A549 cells at 48 h. (h) Microscopic images of A549 cells incubated with poly (I:C) (10 µg/ml) for 48 h. The magnification was 100 ×. The scale bar represents 100 µm. The viability of cells incubated with poly (I:C) was assessed, using LDH assay. (i) The viability of BEAS-2B cells incubated with poly (I:C) for 24 h. (j) The viability of A549 cells incubated with poly (I:C) for 24 h. (k) The viability of BEAS-2B cells incubated with poly (I:C) for 48 h. (l) The viability of A549 cells incubated with poly (I:C) for 48 h. Data are presented relative to the control as mean ± SEM. * $P \leq 0.05$, compared with the control group, using one-way analysis of variance with Bonferroni post-test, N = 3.

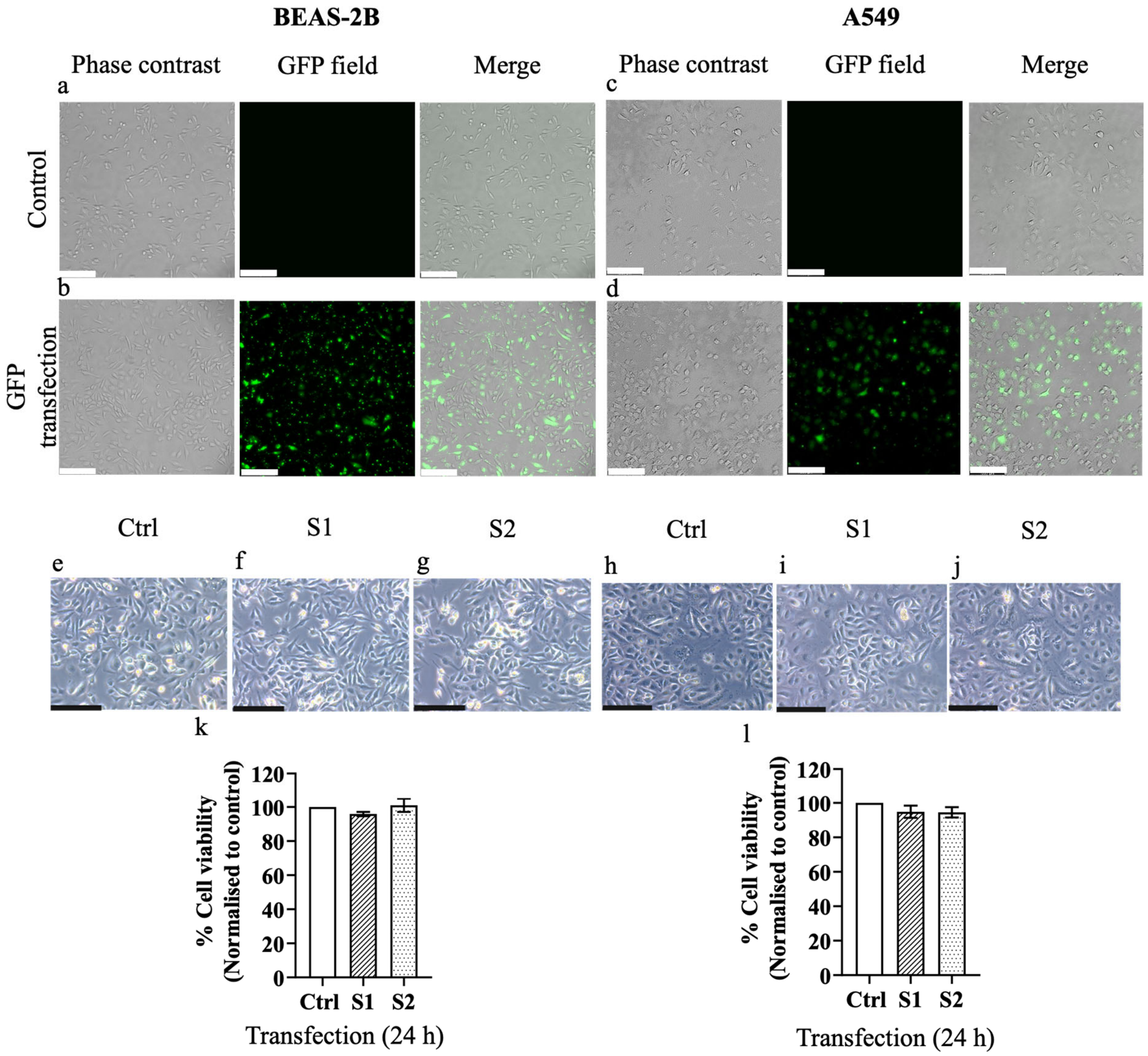

Figure S2. Effect of S1 or S2 transfection on viability of airway epithelial cells. BEAS-2B or A549 cells were cultured in a 24-well plate at 1 × 10^5 cells per well in BEGM or F-12K medium (FBS, 10%), respectively, for 24 h. To confirm transfection efficacy, cells were transfected with GFP (1 µg) for 4 h, washed with PBS and observed under fluorescent microscope at 200 × magnification, N=3. (a) Immunofluorescence images from control BEAS-2B cells. (b) Immunofluorescence images from BEAS-2B cells after GFP transfection. (c) Immunofluorescence images from control A549 cells. (d) Immunofluorescence images from A549 cells after GFP transfection. The scale bar represents 133.7 µm. To assess cell viability, BEAS-2B or A549 cells were transfected with S1 or S2 (1 µg) for 24 h. (e) Light microscopic images of control BEAS-2B cells. (f) Light microscopic images of BEAS-2B cells transfected with S1. (g) Light microscopic images of BEAS-2B cells transfected with S2. (h) Light microscopic images of control A549 cells. (i) Light microscopic images of A549 cells transfected with S1. (j) Light microscopic images of A549 cells transfected with S2. (k) Viability of BEAS-2B cells transfected with S1 or S2 was assessed after 24 h, using LDH assay, N=4. (l) Viability of A549 cells transfected with S1 or S2 was assessed after 24 h, using LDH assay, N=3. Data are presented relative to the control as mean ± SEM.

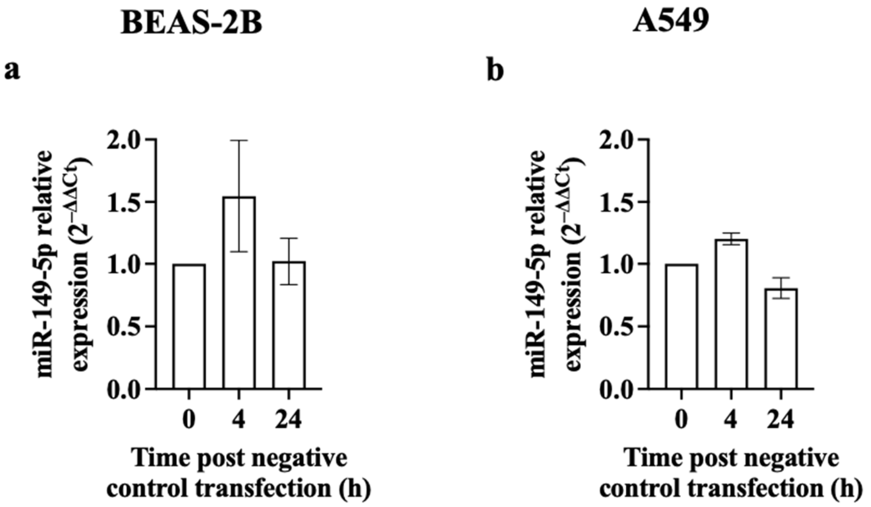

Figure S3. Effect of transfection reagent on miR-149-5p levels in airway epithelial cells. BEAS-2B or A549 cells were cultured in a 24-well plate at 1 × 10^5 cells/well in BEGM or F-12K medium (FBS, 10%) for 24 h, respectively. BEAS-2B or A549 cells were incubated with protein transfection reagent as transfection control for 0, 4 or 24 h. miR-149-5p expression was assessed, using RT-qPCR. (a) miR-149-5p expression in BEAS-2B cells exposed to transfection control, N=5. (b) miR-149-5p expression in A549 cells exposed to transfection control, N=3. The cycle threshold (Ct) value of miR-149-5p was normalized to that of RNU44 (ΔCt). Data are presented relative to the control at baseline (ΔΔCt) as mean ± SEM.

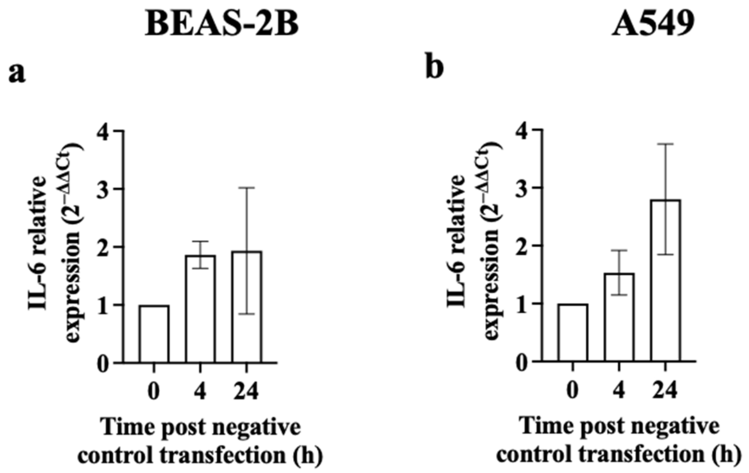

Figure S4. Effect of transfection reagent on *IL-6* mRNA levels in airway epithelial cells. BEAS-2B or A549 cells were cultured in a 24-well plate at a density of 1 × 10^5 cells per well in BEGM or F-12K medium (FBS, 10%) for 24 h, respectively. BEAS-2B or A549 cells were incubated with protein transfection reagent as transfection control for 0, 4 or 24 h. *IL-6* mRNA expression was assessed, using RT-qPCR. (a) *IL-6* mRNA expression in BEAS-2B cells exposed to transfection control, N=3. (b) *IL-6* mRNA expression in A549 cells exposed to transfection control, N=3. The cycle threshold (Ct) value of *IL-6* was normalised to that of 18S rRNA (ΔCt). Data are presented relative to the control at baseline (ΔΔCt) as mean ± SEM.

Table S1. *TLR2* mRNA expression in A549 cell after S1 or S2 transfection. A549 cells were cultured in a 24-well plate at 1 × 10^5 cells per well in F-12K medium (FBS, 10%) for 24 h. A549 cells were transfected with S1 or S2 (1 μg) for 24 h. *TLR2* mRNA levels were assessed at baseline and after S1 or S2 transfection for 24 h using RT-qPCR, N=3. Data are presented as Ct values of *TLR2* or 18S rRNA.

Time post transfection (h)	A549 cells transfected with S1 or S2 subunit		
	N#1		
	Sample name	TLR2 (Ct)	18s rRNA (Ct)
0 h	Baseline control	37.62	11.76
	Baseline control	38.22	11.92
24 h	S1	37.50	10.36
	S1	36.89	10.25
24 h	S2	38.11	11.68
	S2	38.99	11.63
	N#2		
	Sample name	TLR2 (Ct)	18s rRNA (Ct)
0 h	Baseline control	38.18	11.68
	Baseline control	38.89	11.88
24 h	S1	38.34	11.05
	S1	38.55	11.73
24 h	S2	39.36	11.99
	S2	40.00	12.03
	N#3		
	Sample name	TLR2 (Ct)	18s rRNA (Ct)
0 h	Baseline control	40.00	12.70
	Baseline control	39.39	13.35
24 h	S1	38.95	13.25
	S1	40.01	13.34
24 h	S2	37.78	13.69
	S2	39.59	13.56

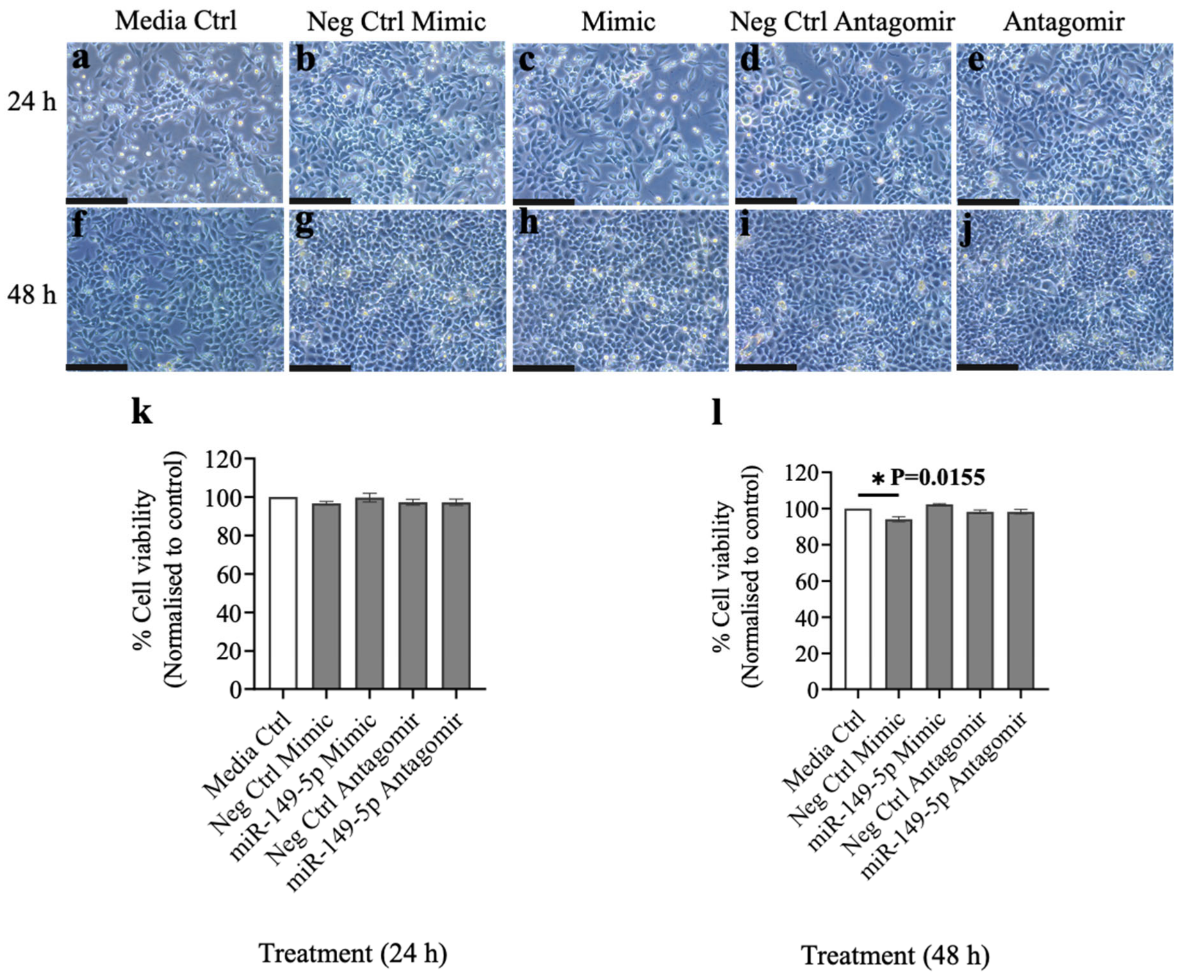

Figure S5. Effect of miR-149-5p mimic or antagomir transfection on the viability of BEAS-2B cells. BEAS-2B cells were transfected with miR-149-5p mimic or antagomir or negative control mimic or antagomir (5 nM) in BEBM (ITS, 1%) for 24 or 48 h. Cell viability was assessed using LDH assay. Light microscopic images of BEAS-2B cells (a) incubated with BEBM (ITS+1, 1%), (b) transfected with negative control mimic, (c) transfected with miR-149-5p mimic, (d) transfected with negative control antagomir, (e) transfected with miR-149-5p antagomir for 24 h, respectively. Light microscopic images of BEAS-2B cells (f) incubated with BEBM (ITS+1, 1%), (g) transfected with negative control mimic, (h) transfected with miR-149-5p mimic, (i) transfected with negative control antagomir, (j) transfected with miR-149-5p antagomir for 48 h, respectively. The magnification was 100 ×. Scale bar: 100 μM. (k) Viability of BEAS-2B cells after transfection with miR-149-5p mimic or antagomir for 24 h was assessed, using LDH assay. (l) Viability of BEAS-2B cells after transfection with miR-149-5p mimic or antagomir for 48 h was assessed, using LDH assay. Data are presented relative to the media control as mean ± SEM. * P ≤ 0.05, compared to the media control, using one-way analysis of variance with Bonferroni post-test, N=3.

Table S2. Effect of miR-149-5p mimic or antagomir transfection on the release of IL-6 in BEAS-2B cells. BEAS-2B cells were cultured in a 24-well plate at 1 × 10^5 cells per well in BEGM for 24 h. BEAS-2B cells were transfected with miR-149-5p mimic or antagomir (5nM) in BEBM (ITS, 1%). IL-6 release was assessed after miR-149-5p mimic or antagomir transfection for 48 h, N=3. The IL-6 standard concentrations were 9.4, 18.8, 37.5, 75, 150, 300 and 600 pg/ml. Samples were measured under the lowest standard 9 pg/ml.

	Transfection	IL-6 release 48 h post transfection (pg/mL)	Lowest IL-6 standard used (9 pg/mL)
N1	Negative control mimic	9.270	
	Mimic	2.783	Out of range
	Negative control antagomir	6.729	Out of range
	Antagomir	6.998	Out of range
N2	Negative control mimic	6.901	Out of range
	Mimic	3.614	Out of range
	Negative control antagomir	4.682	Out of range
	Antagomir	6.969	Out of range
N3	Negative control mimic	4.238	Out of range
	Mimic	4.199	Out of range
	Negative control antagomir	5.942	Out of range
	Antagomir	5.383	Out of range
